# Species and structural diversity of trees at the structural type level

**DOI:** 10.1186/s12862-024-02229-y

**Published:** 2024-03-28

**Authors:** Yuanfa Li, Liting Wei

**Affiliations:** 1 Guangxi Key Laboratory of Forest Ecology and Conservation, College of Forestry, Guangxi University, Nanning, 530004 China; 2 Laibin Jinxiu Dayaoshan Forest Ecosystem Observation and Research Station of Guangxi. No, Jinxiu County, 95 Gongde Road, Laibin, 545700 Guangxi China; 3 College of Forestry, Guangxi University, Daxue East Road 100, Xixiangtang DistrictGuangxi Province, Nanning, 530004 China

**Keywords:** Biodiversity, Differentiation, Distribution pattern, Habitat heterogeneity, Natural forest, Species mixture

## Abstract

**Background:**

Species and structural diversity are important for understanding the formation of forest communities, key ecological processes, and improving forest ecological functions and services, but their spatial characteristics have received little attention. Based on the spatial relationships among neighbouring trees, we proposed to divide trees within a structural unit into 15 structural types, and used the univariate distributions of the uniform angle index (W), mingling (M), and dominance (U), along with four common species diversity indices, to analyse the diversity of structural types in natural forests near the Tropic of Cancer.

**Results:**

Only a portion of clumped class maintained aggregation, most exhibited a random pattern. Species mixture increased exponentially across distribution classes, and abundance and richness exhibited an initial increase followed by a slight decrease. The distribution patterns of mixture classes varied from highly clustered to random, and M distributions gradually shifted from an inverted J-shaped curve to a J-shaped curve. Abundance and richness exhibited an exponential distribution, whereas the Shannon–Wiener index increased linearly. The W distribution of differentiation classes approximated a normal distribution, whereas M distributions exhibited a J shape. The U distribution of each structure type was approximately 0.2.

**Conclusions:**

These results reveal the species and structural diversity characteristics of trees at the structural type level and expand our knowledge of forest biodiversity. The new method proposed here should significantly contribute to biodiversity monitoring efforts in terrestrial ecosystems, and suggests that higher standards for the simulation and reconstruction of stand structure, as well as thinning in near-natural forests, is warranted.

**Supplementary Information:**

The online version contains supplementary material available at 10.1186/s12862-024-02229-y.

## Introduction

Forests provide primary habitats for a variety of species, as well as essential ecosystem services to humans [1]. Climate change, environmental pollution, frequent natural disasters (e.g. drought, freezing, and wildfires), human disturbance, resource exploitation (e.g. logging, grazing, and changing vegetation types), and land use have resulted in the fragmentation and degradation of many forests [2–5]. This has significantly impacted the distribution patterns, species composition, growth, and diversity of forests, thus reducing ecological stability and resistance to disturbance [1, 6]. Forest environment (e.g., soil, microclimate), fauna and subsurface components (e.g. litter) associated with this process have also been substantially impacted [7–10]. Rapid losses in forest biodiversity have attracted much attention and generated considerable concern [6], and numerous nature reserves, among other protected areas, have been established to protect biodiversity and provide models for forest management [11]. An accurate understanding of the quantitative characteristics of biodiversity at different scales would facilitate assessments of forest conservation status, management efforts, and monitoring conservation and restoration efforts.

Biodiversity refers to forest components (species, functional traits, and genes), structure, and functions/processes [12, 13]. Among them, species and structural diversity have emerged as the most important aspects of forest biodiversity [14–17], and play functional roles (e.g. dead wood) in forest ecosystems. Species are the primary component of forest ecosystems, and species composition largely determines forest productivity, spatial allocation, and carbon storage, among other characteristics [18]. Forest structure plays an important role in regulating species composition, seed dispersal, establishment, interactions between neighbouring trees, habitat type, resource use (e.g. light and nutrition), and other ecological processes related to forest growth [16, 19–21]. Forest structure also strongly affects ecological functions (e.g. biomass, carbon storage, and productivity), stress resistance, successional trajectories, the suitability of silvicultural methods, and stand microenvironments [2, 9, 22, 23]. While on a small scale, species and structure, together with other environmental factors (e.g. local climate and soil), directly or indirectly influence forest stability [1, 2]. Most biodiversity studies focus on one or the other, and rarely address both factors simultaneously [9]. Studies considering both species and structural diversity may improve our understanding of the processes and mechanisms underlying forest community development [24].

Both species and structural diversity are related to spatial scale [17, 25]. From small to large, species diversity can be divided into α, β, γ, and δ levels, and each of them has been well documented. α diversity refers to species diversity at the community, stand, or quadrat level [22]. β diversity describes species turnover between communities, whereas γ and δ describe broader scale patterns [26]. The studies of structural diversity mainly focus on the non-spatial properties of diameter class/ontogenetic stages, lifeforms, chronosequences and quadrat [1, 2, 4, 9, 17, 24, 26]. To date, however, few spatial analyses involving species have been applied in biodiversity studies [27], and few studies have highlighted the utility of analysing spatial diversity [14]. Not to mention the relationship between species and structures below the quadrat level.

**Fig. 1:**
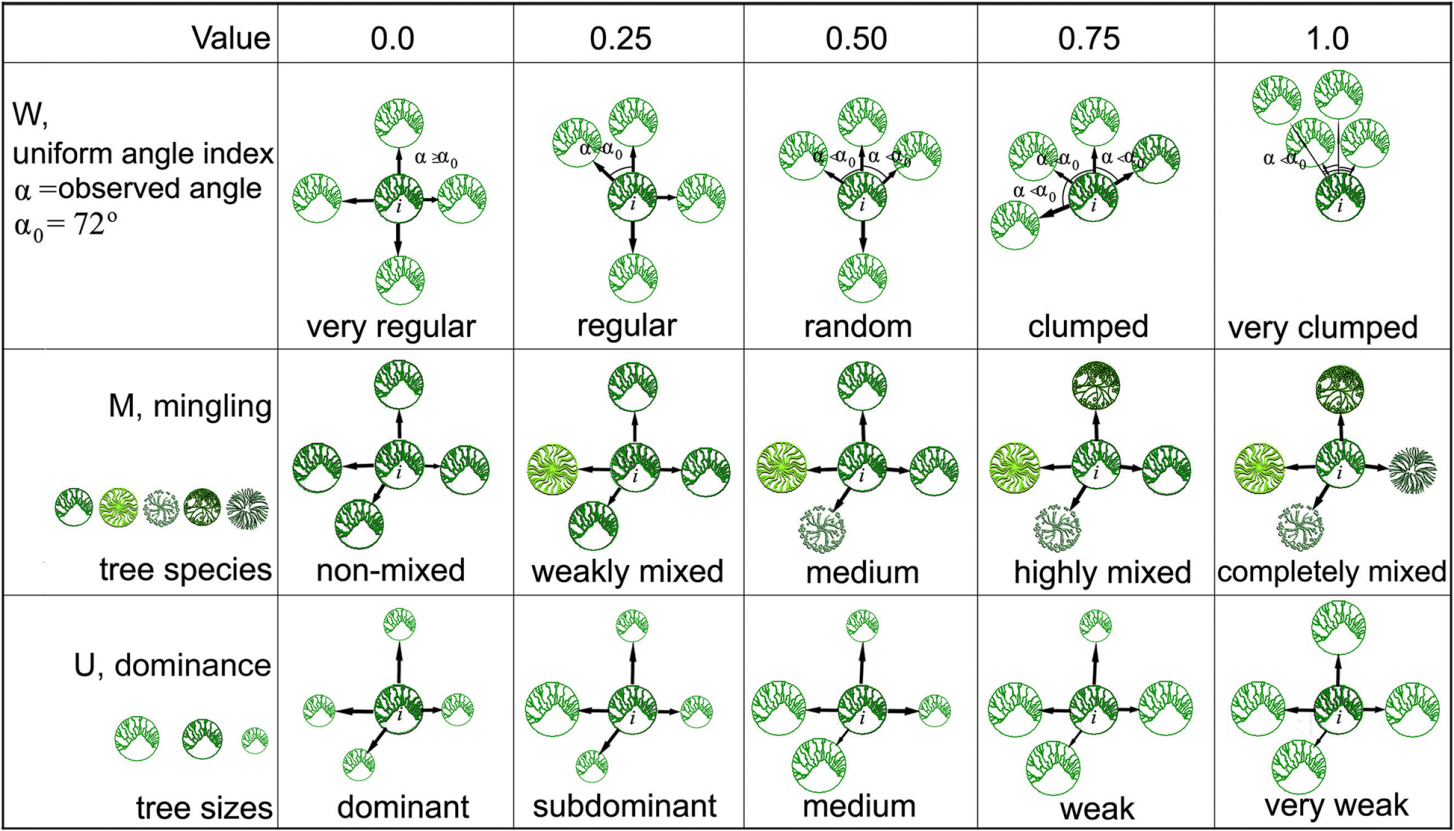
Explanations for stand spatial structural parameters. Structural unit contains a reference trees i and four nearest neighbors and their spatial relationships can be described by uniform angle index (W), mingling (M) and dominance (U), respectively. In the top panels, patterns change from regularity to clump with increasing W value. In the medium panels, reference tree i has more heterospecific neighbors with increasing M value. In the bottom panels, reference tree is less dominant in structural unit with increasing U value

The introduction of stand spatial structure parameters (SSSPs), such as the uniform angle index (W), mingling (M), and dominance (U), has enhanced analyses of spatial structure [17, 28–32]. These metrics describe the distributional attributes of the four nearest neighbouring trees in a structural unit or frame around reference tree *i*, including the degree of species mixing and size differentiation (Fig. 1). They are independent of each other and each index has the same range values [29], which just meets the conditions for tree classification. That is, trees in any quadrat can be further divided into five distribution classes, five mixture classes and five differentiation classes, which represent species groups with different aggregation, mixing and differentiation, respectively. We called them structural types in this study. Obviously, structural type level is smaller than quadrat and allows the analysis of species and structural diversity in space.

Since the properties of structural types have yet to be explored. This study mainly focused on the structural diversity (i.e. tree point distribution, species mixing, and size differentiation) and species diversity of structural types in natural forests. Conspecific aggregation is prevalent in natural forests [33–37], we assumed that the aggregation of conspecifics increases with increasing W value (Fig. 1), that is, the aggregation and interspecific isolation of distribution classes increase while species diversity and size differentiation decrease (Hypothesis 1). While other types of interactions (e.g. facilitative or neutral) in forest ecosystems cannot be ignored, intraspecific exclusion is thought to be more prevalent [33, 38]. Strong intraspecific competition will lead to size differentiation, self-thinning and promotes the formation of species and structural diversity [39]. As a result, the distribution pattern of highly mixed trees may become more uniform and their differentiation is more obvious (Hypothesis 2). Similarly, highly differentiated trees might have a greater mixture and a more uniform pattern (Hypothesis 3). We used dataset from dozens of natural forest plots near the Tropic of Cancer (Guangxi, China) to test these hypotheses and interpreted the results.

## Materials and methods

### Study sites

**Fig. 2:**
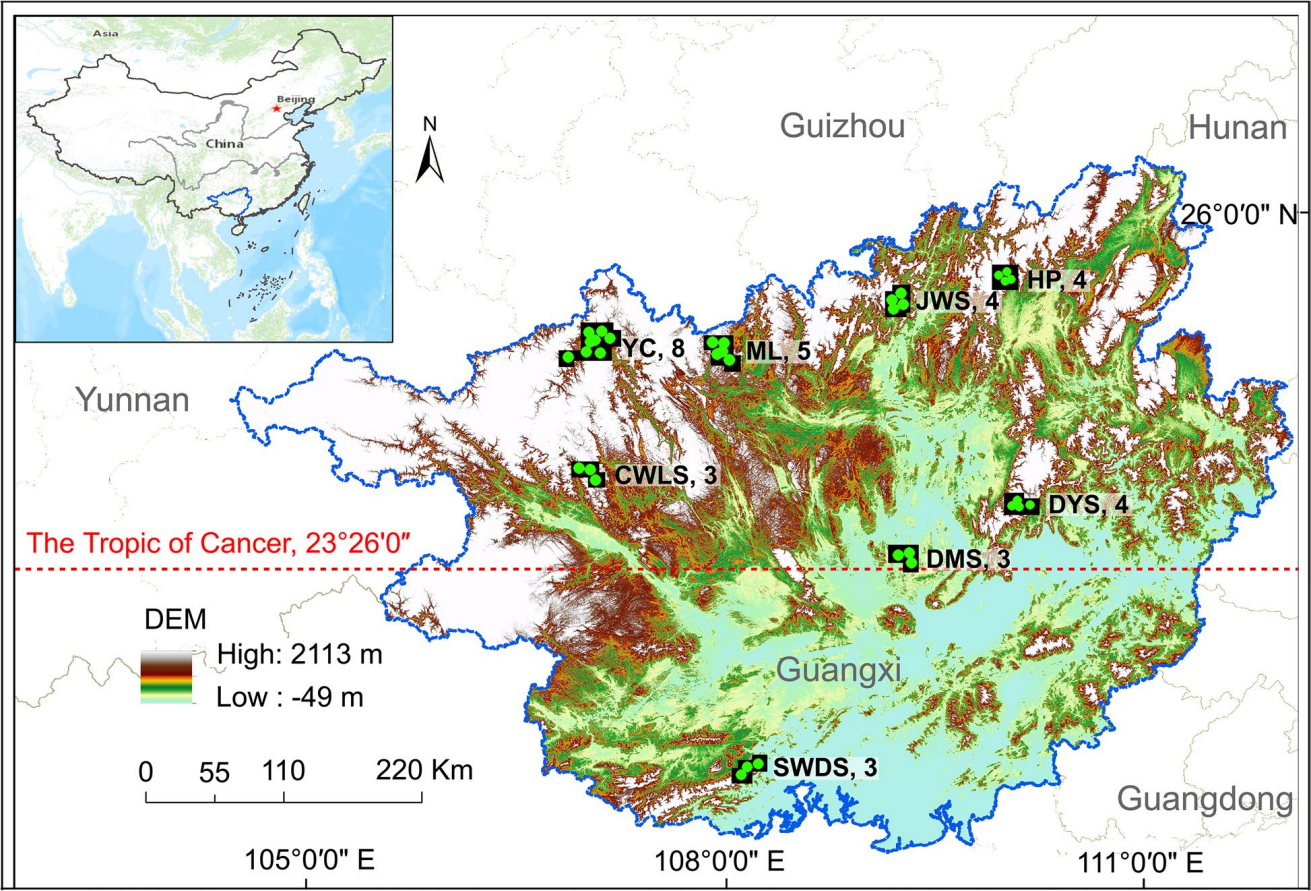
Locations of the study sites. Green dots represent quadrats and Arabic numeral represents the number of quadrat in each reserve. SWDS = Shiwandashan nature reserve, DMS = Damingshan nature reserve, DYS = Dayaoshan nature reserve, CWLS = Cenwanglaoshan nature reserve, ML = Mulun nature reserve, YC = Yachang nature reserve, JWS = Jiuwanshan nature reserve, and HP = Huaping nature reserve

The study sites were located in the primary national nature reserves of Guangxi Zhuang Autonomy Region, China. From south to north, these include the Shiwandashan (SWDS), Damingshan (DMS), Dayaoshan (DYS), Cenwanglaoshan (CWLS), Mulun (ML), Yachang (YC), Jiuwanshan (JWS), and Huaping (HP) reserves. The reserves are located near or on The Tropic of Cancer (23° 26ʹ N) (Fig. 2), and thus represent the northern tropics and southern and mid-subtropics. The region is characterised by abundant rainfall and long, hot summers that last from May to September. Autumn (October–November), winter (December–January), and spring (February–April) are all short and relatively warm. The reserves are distributed throughout the mountains of Guangxi, and support montane forest ecosystems. They are composed of evergreen broad-leaved mixed forests, broad-leaved mixed forests, and evergreen and deciduous broad-leaved mixed forests (Table S1). These forests are highly structurally complex and rich in species, making them a global biodiversity hotspot [1, 17]. The vegetation is mainly composed of secondary forests, with some plantations and a small amount of primary and mature forests. All of the study reserves are dominated by broadleaf trees. Due to strict regulations, there has been little anthropogenic disturbance to the vegetation since the reserves were established.

### Plot establishment

Between 2016 and 2022, 34 standard plots were established in the study reserves. Quadrat locations were selected based on topographical and forest characteristics, including species composition, structure, and growth status (Table 1). All quadrats were established in natural forests representing different developmental stages, including early, mid-, and late-successional. Some stands have a known date of origin, whereas others are poorly documented (Table 1). Nevertheless, all stands represent locally common forest types and have not recently been impacted by disturbance. The quadrats were randomly located on mountains. With the aid of a total station, we first delineated the perimeters of the quadrats and then divided each quadrat into *n* 20 × 20 m quadrats. We measured the coordinates of all trees in each quadrat with a DBH ≥ 1 cm. Each tree was recorded, identified to species (by doctor Lei Wang from College of Forestry, Guangxi University non-doposited), and marked. Furthermore, we noted the growth status of each tree, including characteristics such as stem form, vitality, disease, and insect damage. Information about non-biological habitat attributes (e.g. soil type and thickness, litter, rock, and coarse woody debris), disturbance, and stand age was also collected. Finally, a global positioning system was used to determine the geographical position of each quadrat.

**Table 1:** Basic information of 34 quadrats close to the Tropic of Cancer

Quadrat	Area (m^2^)	R	N	Age (years)	$\overline{{\varvec{D}}{\varvec{B}}{\varvec{H}} }$±SD (cm)	Climate	Habitat	Disturbance
CWLS-1	100 m × 100 m	147	4593	> 200	**6.8** ± 8.5	north tropic	non-karst	no record
CWLS-2	100 m × 100 m	147	3052	> 200	**7.4** ± 10.2	north tropic	non-karst	no record
CWLS-3	100 m × 100 m	179	3627	> 200	**6.1** ± 8.2	north tropic	non-karst	no record
DMS-1	100 m × 100 m	96	5755	> 60	**6.6** ± 5.2	south subtropics	non-karst	no record
DMS-2	100 m × 100 m	87	6614	> 60	**6.1** ± 4.2	south subtropics	non-karst	no record
DMS-3	100 m × 100 m	73	6110	> 60	**7.4** ± 4.6	south subtropics	non-karst	no record
DYS-1	100 m × 100 m	118	4407	> 70	**6.4** ± 6.7	south subtropics	non-karst	no record
DYS-2	100 m × 100 m	124	4117	> 70	**5.6** ± 7.2	south subtropics	non-karst	no record
DYS-3	100 m × 100 m	91	3160	> 70	**7.8** ± 8.2	south subtropics	non-karst	no record
DYS-4	100 m × 100 m	88	4359	> 70	**6.9** ± 8.8	south subtropics	non-karst	no record
HP-1	100 m × 100 m	117	4044	> 40	**7.1** ± 7.6	middle subtropics	non-karst	no record
HP-2	100 m × 100 m	98	6789	> 40	**4.9** ± 5.9	middle subtropics	non-karst	no record
HP-3	100 m × 100 m	124	6636	> 40	**5.1** ± 6.6	middle subtropics	non-karst	no record
HP-4	100 m × 100 m	105	6592	> 40	**5.8** ± 7.3	middle subtropics	non-karst	no record
JWS-1	100 m × 100 m	95	3024	> 55	**6.5** ± 7.8	middle subtropics	non-karst	no record
JWS-2	100 m × 100 m	98	4545	> 55	**6.4** ± 7.2	middle subtropics	non-karst	no record
JWS-3	100 m × 100 m	97	6515	> 55	**6.0** ± 4.2	middle subtropics	non-karst	no record
JWS-4	100 m × 100 m	103	8540	> 55	**6.0** ± 3.8	middle subtropics	non-karst	no record
ML-1	100 m × 100 m	97	3577	> 45	**5.5** ± 5.5	middle subtropics	karst	no record
ML-2	100 m × 100 m	128	4278	> 45	**5.5** ± 4.9	middle subtropics	karst	no record
ML-3	100 m × 100 m	136	5233	> 45	**4.7** ± 4.6	middle subtropics	karst	no record
ML-4	100 m × 100 m	132	3999	> 45	**5.8** ± 5.5	middle subtropics	karst	no record
ML-5	100 m × 100 m	96	3479	> 45	**6.1** ± 4.5	middle subtropics	karst	no record
SWDS-1	100 m × 100 m	129	6690	> 30	**5.4** ± 4.9	middle subtropics	non-karst	no record
SWDS-2	100 m × 100 m	181	8847	> 30	**5.3** ± 3.8	middle subtropics	non-karst	no record
SWDS-3	100 m × 100 m	155	7990	> 30	**5.5** ± 4.4	middle subtropics	non-karst	no record
YC-1	100 m × 100 m	18	2391	30	**11.5** ± 6.7	middle subtropics	non-karst	no
YC-2	80 m × 80 m	22	1895	57	**8.9** ± 8.6	middle subtropics	non-karst	very slight
YC-3	100 m × 80 m	47	3050	> 100	**16.1** ± 14.5	middle subtropics	non-karst	selective harvest
YC-4	100 m × 60 m	27	1743	57	**8.6** ± 8.1	middle subtropics	non-karst	very slight
YC-5	80 m × 70 m	35	1074	57	**10.3** ± 8.4	middle subtropics	non-karst	very slight
YC-6	200 m × 110 m	55	4596	> 300	**7.2** ± 10.4	middle subtropics	karst	no record
YC-7	200 m × 110 m	72	6497	> 60	**8.1** ± 7.4	middle subtropics	karst	selective harvest
YC-8	200 m × 80 m	86	9815	> 300	**5.8** ± 8.2	middle subtropics	non-karst	no

### Data analyses

We calculated the parameters for each tree in all quadrats [29], and divided the results into 15 structural types (5 each for W, M, and U) using thresholds of 0.0, 0.25, 0.50, 0.75, and 1.00 to delineate the classes (Fig. 1). Correspondingly, structural types refer to the various levels of W_*i*_, M_*i*_ and U_*i*_, which we refer to as distribution classes, mixture classes, and differentiation classes, respectively (Fig. 1, Table 2). We then calculated the parameters for each structural type, and considered their univariate distributions as metrics of spatial diversity [28, 29]. We used the *wilcox.test* function to test for significant differences among the univariate distributions. Because the mean value of W is substantially influenced by the number of individuals (N) per quadrat, we calculated 95% confidence intervals for each structural type using the formula ${{\text{y}}}_{0.05}=0.5\pm 1.96\upsigma \times 0.21034{N}^{-0.48872}$ [40], and considered the result to represent the range of random distribution. We then determined whether trees were distributed in an aggregated, random, or regular fashion. For better explain and compare the results with other studies, we further considered W_*i*_ = 0.00 and 0.25 as regular class (ReC), W_*i*_ = 0.50 as random class (RaC), and W_*i*_ = 0.75 and 1.00 as clumped class (CC). Similarly, we considered M_*i*_ = 0.00 and 0.25 to be low-mixture class (LMC), M_*i*_ = 0.50 to be medium-mixture class (MMC), and M_*i*_ = 0.75 and 1.00 to be highly mixed class (HMC), and U_*i*_ = 0.00 and 0.25 as dominant class (DC), U_*i*_ = 0.50 as moderately dominant class (MDC), and U_*i*_ = 0.75 and 1.00 as weak (non-dominant) class (WC).

**Table 2:** Stand spatial structure parameters, species diversity indices and similarity index used in this study

Indices	Formula	Explanation	References
SSSPs	${{\text{W}}}_{i}=\frac{1}{4}\sum_{j=1}^{4}{Z}_{ij}$	When the *j*^th^ angle $\alpha$ is smaller than the *i*^th^ standard angle ${\alpha }_{0}$=72°, *z*_*ij*_ is equal to 1. Or, *z*_*ij*_ is 0	[29]
	${{\text{U}}}_{i}=\frac{1}{4}\sum_{j=1}^{4}{K}_{ij}$	When the reference tree *i* is smaller than the neighbor tree *j*, *k*_*ij*_ is equal to 1. Or, *k*_*ij*_ is 0	[29]
	${{\text{M}}}_{i}=\frac{1}{4}\sum_{j=1}^{4}{V}_{ij}$	When the neighbor *j* is not the same species as the reference tree *i*, *v*_*ij*_ is equal to 1. Or, *v*_*ij*_ is 0	[29]
SDI	${{\text{H}}}^{\mathrm{^{\prime}}}= -{\sum }_{i=1}^{{\text{S}}}{p}_{i}{\text{ln}}({p}_{i})$	Hʹ = Shannon–Wiener index, *S* = number of species, *p*_*i*_ = proportion of individuals in the *i*^th^ species	[14]
	${{\text{E}}}_{{\text{H}}}=\frac{-\sum {p}_{i}{\text{log}}{p}_{i}}{{\text{ln}}S}$	${{\text{E}}}_{{\text{H}}}$= Pielou evenness index, *S* = number of species, *p*_*i*_ = proportion of individuals in the *i*^th^ species	[14]
SI	${K}_{j}=\frac{j}{({\text{a}}+{\text{b}}-j)}$	${K}_{j}$= Jaccard similarity coefficient,* j* = number of common species between two grades of SSSPs, a, b = number of species only occurred in each grade,* K*_*j*_ ≤ 0.5 represents dissimilarity, *K*_*j*_ > 0.5 represents similarity	[41]

Explanations of SSSPs are showed in Fig. 1. *SSSPs* stand spatial structure parameters, *SDI* species diversity indices, *SI* similarity index

We quantified the species diversity of each structural type using two basic diversity indices: species richness (SR) and N. (number of individual) Furthermore, we calculated an index that describes the relationship between SR and N (i.e. the Shannon–Wiener index; Hʹ) and a second index that characterises the consistency of N among species (i.e. the Pielou evenness index; E_H_) (Table 2). These indices were calculated using the ‘vegan’ package in R [42]. The *wilcox.test* function was used to test for significant differences in species diversity among structural types, and Jaccard’s similarity coefficient and Venn diagrams were used to describe correlations in species composition (Table 2).

## Results

### Spatial diversity of distribution classes

**Fig. 3:**
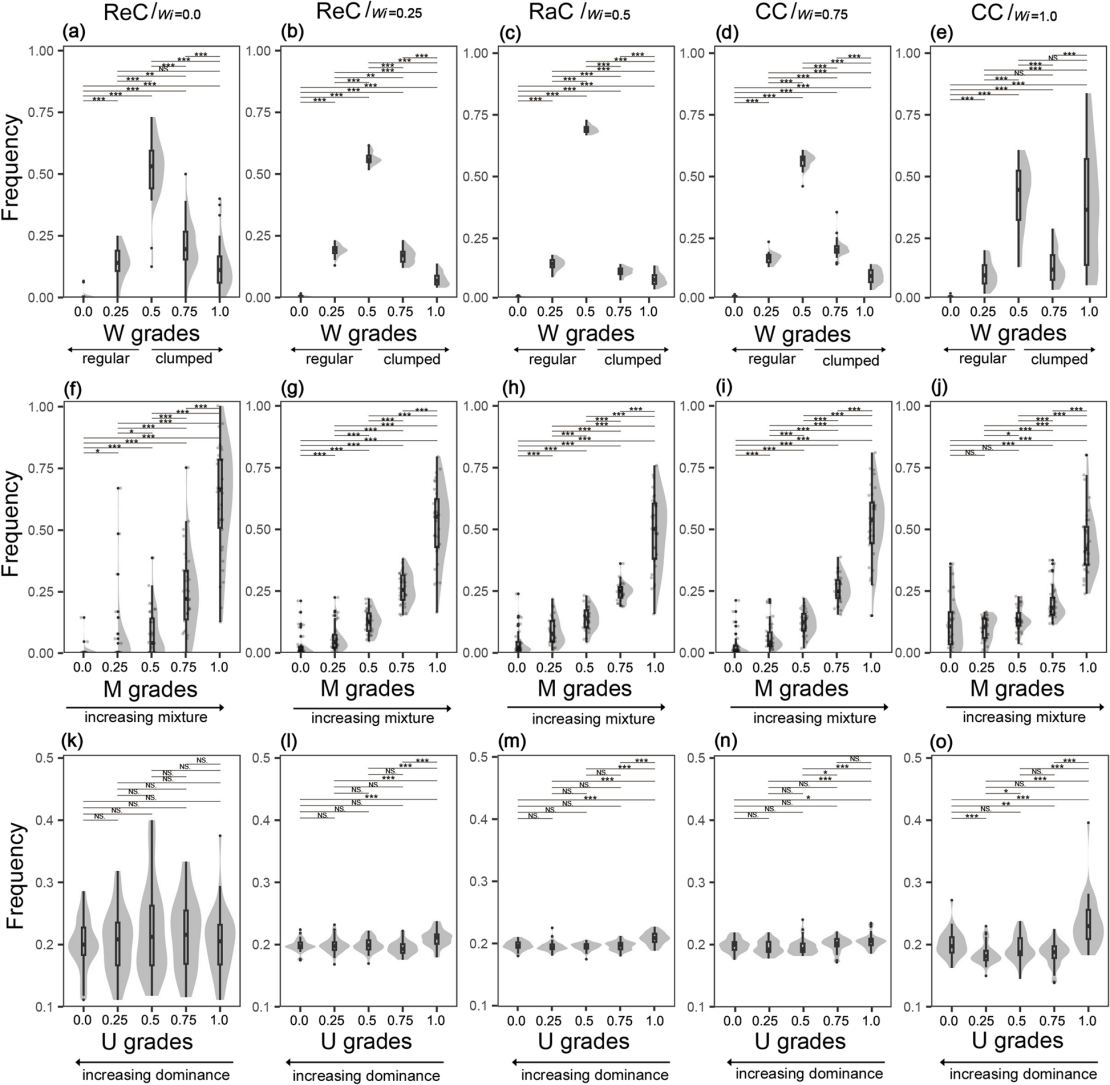
W distribution (**a**-**e**), M distribution (**f**-**j**) and U distribution (**k**-**o**) of distribution classes. ReC = regular class, RaC = random class, CC = clumped class, W = uniform angle index, M = mingling, U = dominance. ***, **, * and NS dominate *p *< 0.001, *p* < 0.01, *p* < 0.05 and *p* ≥ 0.05, respectively

The W distribution of ReC, RaC, and half of CC (W = 0.75) exhibited unimodal patterns, that is, the values first increased with increasing W and then decreased (Fig. 3a–d). The univariate distributions of the other CC group exhibited an initial increase, then decreased before increasing again, and values were relatively variable in the mid and high W grades (Fig. 3e). Across all five distribution classes, trees had significantly smaller values when W was low (*p* < 0.001). M distributions had a similar pattern that increased exponentially, and their mean values gradually decreased from 0.83 to 0.63 (Fig. 3f–j). Distribution classes had similar U distributions, with a mean value of approximately 0.2. ReC exhibited broader distributions than RaC and CC (Fig. 3k–o).

### Spatial diversity of mixture classes

**Fig. 4:**
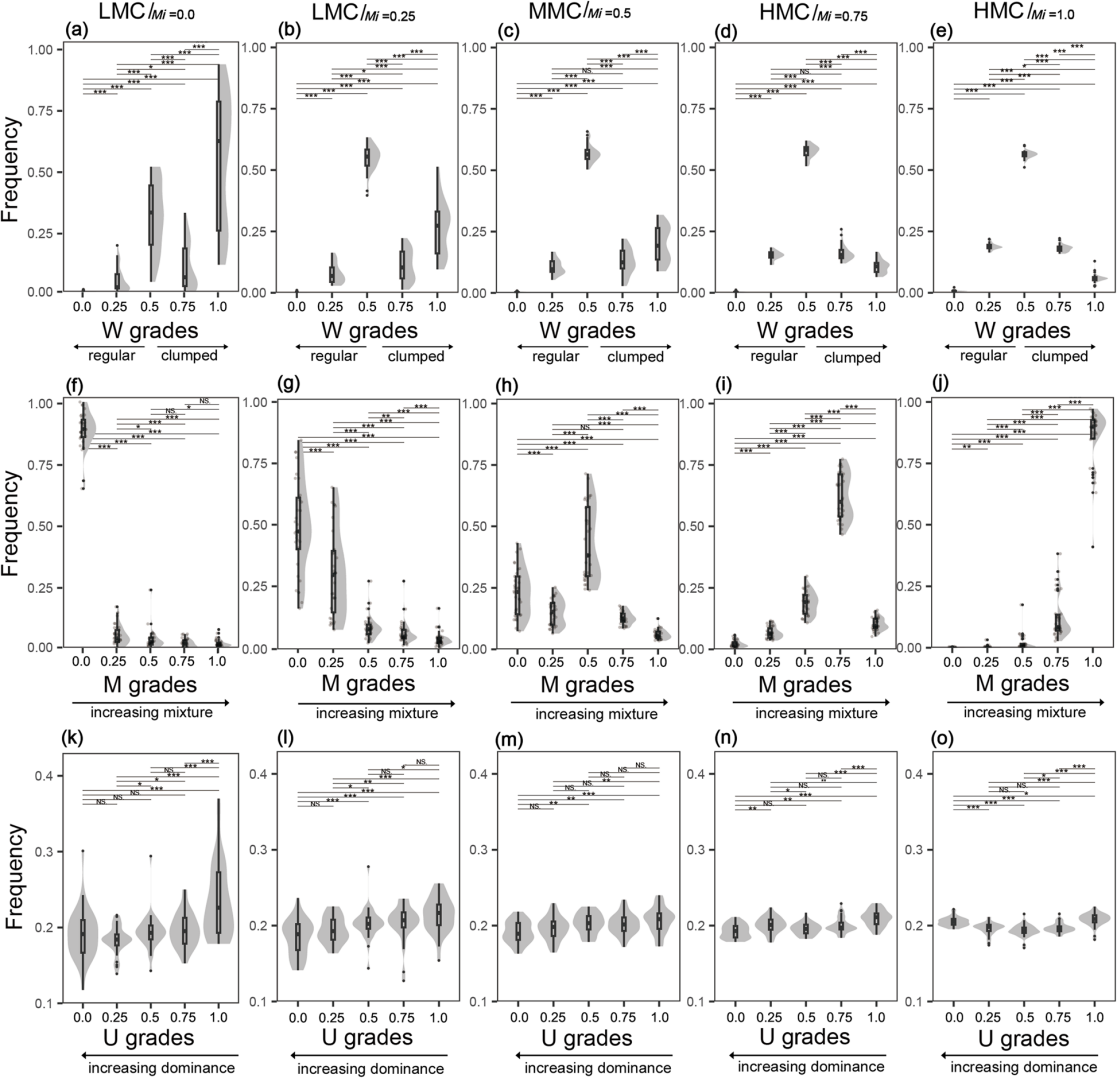
W distribution (**a**-**e**), M distribution (**f**-**j**) and U distribution (**k**-**o**) of mixture classes. LCM = low-mixture class, MMC = medium-mixture class, HMC = highly mixed class, W = uniform angle index, M = mingling, U = dominance.***, **, * and NS dominate *p *< 0.001, *p *< 0.01, *p *< 0.05 and *p *≥ 0.05, respectively

The W values of the low-mixture class exhibited a positive trend, peaking at 0.93 at W = 1.0, with a coefficient of variation as high as 1.22 (Fig. 4a). In the remaining classes, W distributions gradually approximated a normal distribution, and distributions became increasingly narrow (Fig. 4b–e). The M distribution changed substantially among different mixture classes, shifting from an inverted J-shaped to J-shaped distribution, and the mean value increased from 0.05 to 0.97 (Fig. 4f–j). The five mixture classes exhibited similar U distributions, with mean values of approximately 0.2 (Fig. 4k–o).

### Spatial diversity of differentiation classes

**Fig. 5:**
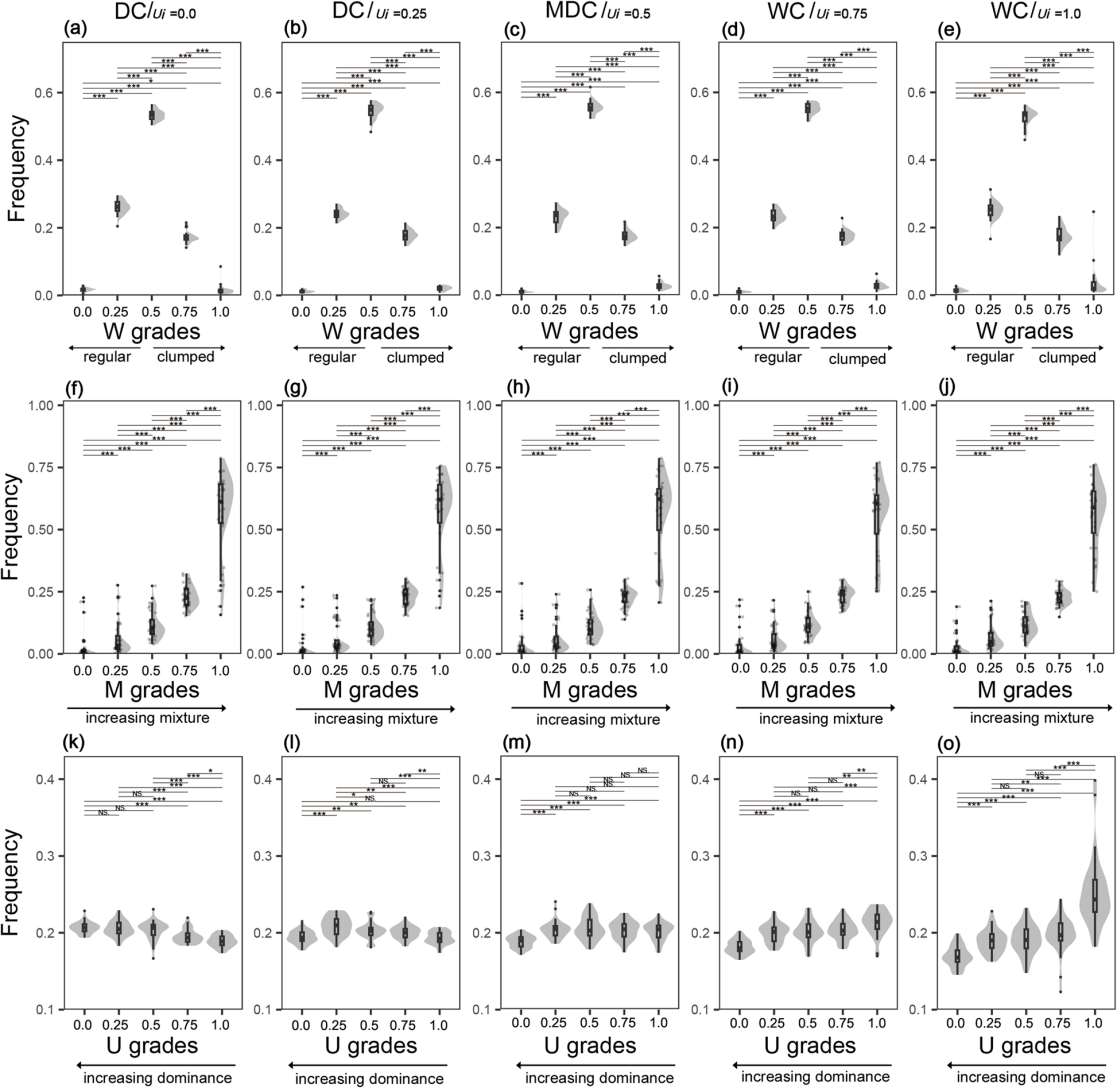
W distribution (**a**-**e**), M distribution (**f**-**j**) and U distribution (**k**-**o**) of differentiation classes. DC = dominant class, MDC = moderately dominant class, WC = clumped class, W = uniform angle index, M = mingling, U = dominance. ***, **, * and NS dominate *p *< 0.001, *p *< 0.01, *p *< 0.05 and *p *≥ 0.05, respectively

All differentiation classes exhibited similar, narrow W distributions that approximated a normal distribution. Mean values increased from 0.01–0.03 at W = 0.0 to 0.20–0.30 at W = 0.25 and 0.50–0.57 at W = 0.50, and then decreased to 0.14–0.22 at W = 0.75 and 0.00–0.09 at W = 1.00 (Fig. 5a–e). M distributions exhibited a slow initial increase followed by a rapid increase, and values increased from around 0.02 to 0.56 (Fig. 5f–j). M grades were similar across differentiation classes, but values differed significantly between M grades (*p* < 0.01). Aside from the U values of WC, which increased from 0.17 to 0.25, other differentiation classes exhibited relatively stable U values of approximately 0.2 (Fig. 5k–o).

### Species diversity among structural types

**Fig. 6:**
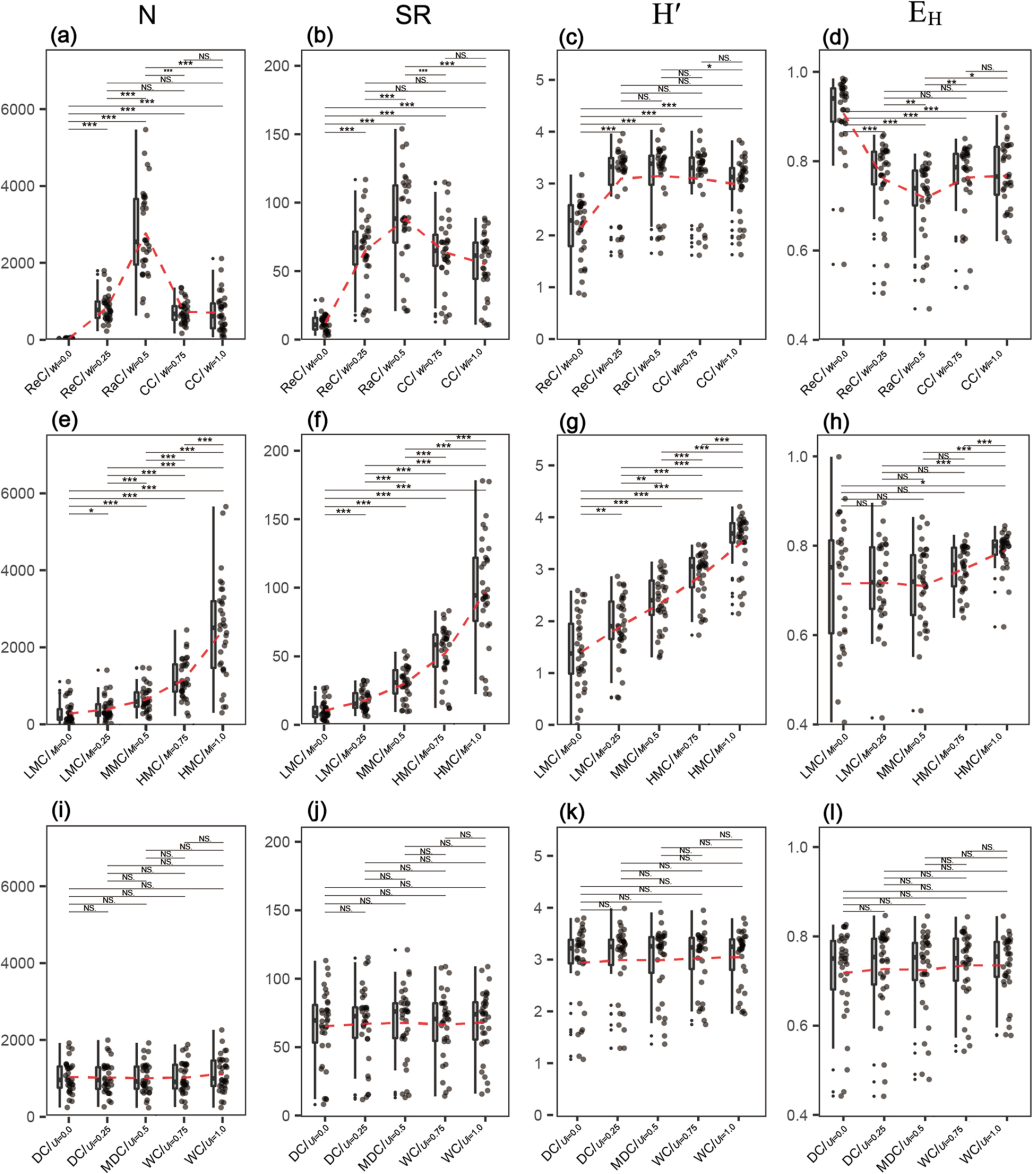
Species diversity of distribution classes (**a**-**d**), mixture classes (**e**-**h**) and differentiation classes (**i**-**l**). ReC = regular class, RaC = random class, CC = clumped class, LMC = low-mixture class, MMC = medium-mixture class, HMC = highly mixed class, DC = dominant class, MDC = moderately dominant class, WC = weak class, N = number of individual, SR = species richness, Hʹ = Shannon-Wiener index, EH = Pielou’ evenness index. ***, **, * and NS dominate *p *< 0.001, *p *< 0.01, *p *< 0.05 and *p *≥ 0.05, respectively

**Fig. 7:**
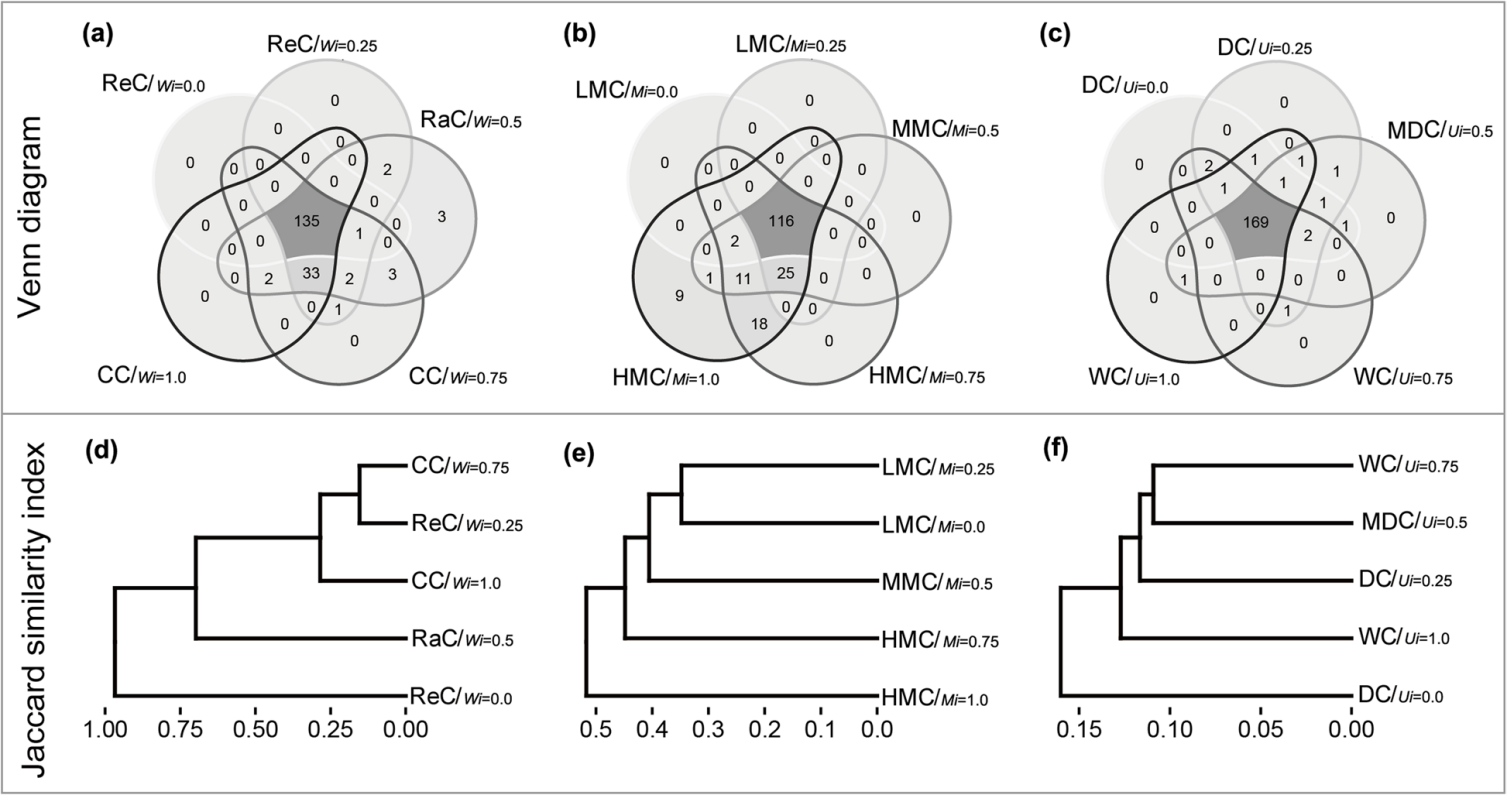
Venn diagrams (**a**-**c**) and Jaccard similarity coefficients (**d**-**f**) of structural types. ReC = regular class, RaC = random class, CC = clumped class, LMC = low-mixture class, MMC = medium-mixture class, HMC = highly mixedclass, DC = dominant class, MDC = moderately dominant class, WC = weak class

As W increased, the mean value of N increased from 22.6 to 2,764.3 and then decreased to 695.03; the differences among structural types were significant. RaC accounted for a much higher proportion of trees than the other classes (Fig. 6a). SR exhibited a similar trend, with a mean that increased from 12.21 to 88.09 and then decreased to 55.24 (Fig. 6b). The mean value of Hʹ increased from 2.17 to 3.16 for ReC and remained high in the remaining structural types (Fig. 6c), whereas E_H_ exhibited the opposite trend (Fig. 6d). N and SR exhibited exponential increases with increased mixture, whereas Hʹ exhibited a significant linear increase (Fig. 6e–g). The E_H_ of HMC was slightly higher and more narrowly distributed than that of the other classes (Fig. 6h). With respect to differentiation classes, the mean values of N, SR, Hʹ, and E_H_ were approximately 1,000, 67, 3.0, and 0.73, respectively (Fig. 6i–l). We observed little variation in SR among differentiation classes, except that the regular and low-mixed classes were both characterised by relatively low SR (Fig. 7a–c). Jaccard similarity coefficients for distribution classes and mixture classes ranged from 0.15–0.99 to 0.35–0.52, while those for the differentiation classes were < 0.16 (Fig. 7d–f).

## Discussion

Although the concept and characterisation of biodiversity have been widely discussed [12, 13, 16, 43], surprisingly little is known about the spatial characteristics of biodiversity at forest stand scales [31, 44]. In many montane tropical and subtropical forests, even small forest patches may exhibit high structural complexity and a range of growing conditions [45], and biodiversity at each scale is very high [15, 25, 36, 38].

### Distribution patterns at the structural type level

In undisturbed or lightly disturbed natural forests, the univariate distribution of uniform angle index is unimodal, which represents a stable pattern [21, 39, 46]. Similar patterns were observed when we classified distribution classes and mixture classes into different structural types (Figs. 3a-d and 4d-e), thereby highlighting stable pattern occur at the stand and sub-stand levels. Random framework (framework when W_*i*_ = 0.5) comprises the majority of basal area and abundance in this pattern, and has a competitive advantage compared with other frameworks and is considered crucial for community stability [27]. Residual classes maintained an evident clumped pattern (Table S2), and most clumps comprised the same species, contributed substantially to stand-level aggregation. This implies that, in stands with high tree density, the average scale of aggregation is much larger than a single structural unit. Various biological and abiotic factors, such as habitat heterogeneity, habitat filtering, gap dynamics, niche differentiation, resource management, stress, and human disturbance, can induce aggregation together [4, 34, 36, 37, 47–49]. The changes of aggregation with distribution classes and mixture classes (Figs. 3a-d and 4a-e) are consistent with Hypothesis 1 and Hypothesis 2, suggesting that distribution patterns can be predicted by species mixture [50], which provides a new approach for the study of distribution patterns.

### Species mixture at the structural type level

The mingling distribution of distribution classes is consistent with that of other natural forests [51], suggesting that hierarchical prediction can be applied to species mixture. In general, species mixture was negatively correlated with aggregation (Fig. 3f-j), while it has a linearly positive correlation with tree sizes [39], indicating that small trees preferred intraspecific aggregation, while large trees preferred interspecific mixing and dispersion, consistent with the mingling-size hypothesis [48, 52, 53]. Many other point pattern studies have shown that aggregation is higher among small and understorey trees compared to large and canopy trees [15, 54, 55], supporting Hypothesis 1. The relationships among distribution pattern, species mixture and tree size can be explained by dispersal limitation and conspecific negative density effects. The way of seeding and regeneration in natural forests largely determines the aggregation of small, conspecific trees, and then intraspecific exclusion and host (disease and insect) invasion lead to mortality and xenogenetic settlement, thus pushing the community towards a more regular pattern comprising multiple species [4, 21, 48]. These effects are important mechanisms by which species coexistence and diversity are maintained in many temperate, tropical, and subtropical forests [38, 49, 56, 57]. In particular, significant habitat heterogeneity near the Tropic of Cancer may enhance aggregation, asymmetric competition and negative density-dependent mortality [37, 47].

The species mixture of mixture classes was consistent with that of the quadrat. Low-mixture class means reference tree *i* and its neighbours are, by definition, conspecifics (Fig. 1). The frequency distribution of this class was opposite to that of other natural forests [32, 39, 51, 58], implies that tree populations are distributed in patches. Highly mixed class, on the contrary, dominated the number of plants in quadrants, and was often surrounded by heterospecifics (Fig. 4i-j), which may be the direct reason for the high mixing of natural forests near the Tropic of Cancer. Its mixture distribution was similar to that of other species-rich natural forests, but with a larger means, indirectly reflecting the study forests were in a highly mixed state [17]. Medium-mixture class is a transitional type, its neighbour could be conspecific or heterospecific (Figs. 1 and 4h). The distribution of mixture classes shows the change of species relationship in detail.

### Size differentiation at the structural type level

There is a negative exponential relationship between dominance and different DBH classes [39], therefore, dominance values represent the advantage of differentiation classes at the stand level. Differentiation classes divided forests near the Tropic of Cancer into groups of trees that were very close to each other in abundance and structural diversity (Fig. 5), but completely different in dominance (Fig. 1) and species composition (Fig. 7f). Their structural diversity approximated those of typical species-rich natural forests [21, 32, 46]. This indicates that trees of different sizes have similar distribution patterns and mixtures at the structural type level, and that these characteristic can be extrapolated to the stand level. The results are contrary to Hypothesis 3, because tree sizes within structural units are relative, and differentiation occurs frequently. Differentiation may occur between conspecifics or heterospecifics, among trees of a particular size class, or between small and large trees, separating the distribution pattern and species mixture of trees of different sizes almost evenly. Distribution classes and mixture classes had similar dominance distributions (Figs. 3k-o, and 4k-o), further emphasizes the fact. Most populations and trees of different DBHs in pine-oak mixed forests and Koran pine broadleaved forests are nearly randomly distributed, and the dominance is only weakly related to uniform angle index [39, 58, 59], which strongly supports our results. Without obvious disturbance, a balanced size differentiation emerges very early [39], which undoubtedly reduce direct competition, thus maximizing the use of three-dimensional spatial resources by the stand. This strategy may facilitate maintenance of species diversity in line with niche differentiation theory [39, 60]. However, large differences in the size of neighbours may obscure the relationship between tree size and spatial structure [61], and stands with different DBH distributions may have similar values of dominance [30]. Dominance and DBH distribution quantify the sizes of tree within different scales, driving forces and community differences may lead to inconsistent results.

### Species diversity at the structural type level

Random class includes the major species of the stand, has high diversity, highlighting its critical role in maintaining community stability [27]. Clumped class also includes numerous species and exhibits high diversity, consistent with the biodiversity characteristics of tropical and subtropical forests [15]. Especially, these natural forests typically contain numerous rare species that have small tree sizes and tend to aggregate [34, 36, 37], thereby enhancing community diversity, evenness and aggregation [17]. Natural forests near the Tropic of Cancer contain karst and non-karst sites that are rich in microhabitats [17] and may promote the formation and aggregation of species diversity [41, 45]. Along bioclimatic gradients across Europe, increased SR alters interspecific and intraspecific relationships, and generally increases the intensity of tree aggregation at small scales [4], which supports our results. Higher functional diversity allows individuals to grow in close proximity, thus promoting the complementary use of resources [4]. The low species diversity of regular class suggests that it plays a minor role in our study stands (Fig. 6a-c). In fact, regular distributions can only occur within a small range, as habitats and germplasm resources are variable in time and space. Few reports of regular distributions in natural forests are available.

Most species and individuals were highly mixed and exhibited high evenness, consistent with findings for tropical broadleaf forests in central Vietnam [32]. Some old-growth forests in temperate regions also exhibit similar characteristics [62]. A high degree of isolation reduces intraspecific competition and is beneficial for species coexistence and the maintenance of biodiversity [4, 33, 48]. By contrast, low-mixture class was characterized by variable evenness and low abundance, richness, and Shannon–Wiener diversity, suggesting intraspecific aggregation. Potential conspecific competition drives community succession [39]. The relationship between species diversity and mixture can be modelled using exponential or linear functions. [63] integrated the number of tree species per structural unit into simple mingling formula (Table 2), and [31] integrated the stand spatial structure parameters into diversity indices to evaluate tree spatial diversity. Together with our results, these studies demonstrate that species diversity is closely related to the structure of neighbouring trees.

Differentiation classes had similar levels of species abundance and richness, which explains the high similarity in structural diversity. Hʹ and E_H_ were evenly separated; however, the variability among dominant class was significantly greater than that of weak class. This may be related to the species composition and successional stage of the community. Some old-growth forests have plenty of dominant populations (e.g., YC-8), while early successional communities have low species richness with respect to dominant canopy species (e.g., YC-2, YC-4 YC-5), and slow growing trees require a considerable amount of time to reach the canopy. Conversely, this pattern also indicates that weak class promotes community diversity [6, 15, 64], which in turn suggests that small trees may decrease diversity as they grow into large trees. Close relationship between species diversity and tree size has been found in natural forests around the Tropic of Cancer [17], which strongly supports our results. At the regional scale, however, the relationship between tree size and species diversity may become increasingly random or inverse [65, 66]. Moreover, evenness is much less important than richness for maintaining biodiversity [2]. It is less stable than other diversity indices, suggesting that the interspecific and individual relationships in natural forests are never in equilibrium.

## Conclusion

A new method based on the spatial relationship of location, mixture and differentiation of neighbouring trees, is proposed for the classification of tree groups. The method classifies quadrats into 15 structural types to enable spatial analysis of species and structural diversity, different from the way of tree attributes (e.g., population, diameter, stratification, life history stage) or combinations of the spatial structure parameters. We further analysed the biodiversity of structural types in main forest ecosystems near the Tropic of Cancer, and found that differentiation classes contribute equally to species and structural diversity. The diversity approaches to quadrat level and are almost independent of tree size and species. The structural diversity of distribution classes and mixture classes exhibits a clear pattern in which small size of conspecifics favour aggregation while big size of heterospecifics favour random distributions. Relationships between tree size, mixture and distribution pattern are close, and can be well explained by popular forest ecology theories in current. The positive relationship between species diversity and mixture is also very close, and could be predicted by generalized linear model. The new method proposed here greatly contributes to biodiversity monitoring and assessment, and suggest that higher standards for the simulation and reconstruction of stand structure, as well as the thinning of near-natural forests, are warranted. With respect to methods, structural types can be divided into smaller groups, and the characteristics of tree species groups after multiple classifications should be explored in future.

### Supplementary Information

**Supplementary Material 1. **

**Supplementary Material 2. **

## Data Availability

The datasets used and/or analysed during the current study are available from the corresponding author on reasonable request.

## Abbreviations

W: Uniform angle index
M: Mingling
U: Dominance
SSSPs: Stand spatial structure parameters
SWDS: Shiwandashan nature reserve
DMS: Damingshan nature reserve
DYS: Dayaoshan nature reserve
CWLS: Cenwanglaoshan nature reserve
ML: Mulun nature reserve
YC: Yachang nature reserve
JWS: Jiuwanshan nature reserve
HP: Huaping nature reserve
DBH: Diameter at breast height
N: Number of individuals
ReC: Regular class
RaC: Random class
CC: Clumped class
LMC: Low-mixture class
MMC: Medium-mixture class
HMC: Highly mixed class
DC: Dominant class
MDC: Moderately dominant class
WC: Weak class
SR: Species richness
Hʹ: Shannon-Wiener index
E_H_: Pielou evenness index
