# Supplementary material for: Species and structural diversity of trees at the structural type level
Source: BMC Ecol Evol. 2024 Mar 28;24:40. doi: 10.1186/s12862-024-02229-y (PMC10976781; doi:10.1186/s12862-024-02229-y)
Supplement: Supplementary file 1 — Supplementary Material 1. [file 12862_2024_2229_MOESM1_ESM.pdf]

Table S1: Main species occur in 8 national natural reserves of Guangxi

No.	SWDS	DMS	DYS	CWLS	YC	ML	HP	JWS
1	Acer coriaceifolium Lévl.	Acer tutcheri Duthie	AcerbicolorF. Chun var.serratifolium(Fang) Fang	Acer davidii Franch.	Quercus fabri Hance	Acer lucidum Metc.	Acer davidii Franch	Acer tutcheri Duthie
2	Acer wilsonii Rehd.	Adinandra bockiana E. Pritzel ex Diels	Adina pilulifera (Lam.) Franch. ex Drade	Acer flabellatum Rehder	Ailanthus altissima (Mill.) Swingle	Adenanthera pavonina Linn. var. microsperma (Teijsm. et Binnend.) Nielsen	Acer fabri Hance	Adenanthera pavonina Linn. var. microsperma (Teijsm. et Binnend.) Nielsen
3	Acronychia pedunculata (Linn.) Miq.	Adinandra glischroloma Hand.-Mazz.	Adinandra millettii (Hook. et Arn.) Benth. et Hook. f. ex Hance	Acer miyabei Maxim.	Ribes burejense F. Schmidt	Aidia cochinchinensis Lour.	Acer tutcheri Duthie	Aidia cochinchinensis Lour.
4	Adina pilulifera (Lam.) Franch. ex Drade	Aidia cochinchinensis Lour.	Aidia cochinchinensis Lour.	Acer sinense Pax	Kalopanax septemlobus (Thunb.) Koidz.	Alangium chinense (Lour.) Harms	Adinandra bockiana E. Pritzel ex Diels	Alniphyllum fortunei (Hemsl.) Makino
5	Adinandra bockiana E. Pritzel ex Diels	Antidesma japonicum Sieb. et Zucc.	Aidia pycnantha (Drake) Tirveng.	Alangium chinense (Lour.) Harms	Broussonetia papyrifera (L.) L'Hér. ex Vent.	Alchornea trewioides (Benth.) Muell. Arg.	Aidia cochinchinensis Lour.	Antidesma japonicum Sieb. et Zucc.
6	Adinandra hainanensis Hayata	Beilschmiedia tsangii Merr.	Alniphyllum fortunei (Hemsl.) Makino	Alangium kurzii Craib	Schima wallichii (DC.) Korth.	Alleizettella leucocarpa (Champ. ex Benth.) Tirvenz	Ailanthus altissima (Mill.) Swingle	Archidendron lucidum (Benth.) Nielsen
7	Aidia cochinchinensis Lour.	Camellia melliana Hand.-Mazz.	Antidesma japonicum Sieb. et Zucc.	Beilschmiedia fordii Dunn	Toona ciliata Roem.	Antidesma japonicum Sieb. et Zucc.	Alangium chinense (Lour.) Harms	Ardisia caudata Hemsl.
8	Alchornea trewioides (Benth.) Muell. Arg.	Castanopsis fabri Hance	Archidendron clypearia (Jack.) Nielsen	Callicarpa bodinieri H. Lév.	Archidendron clypearia (Jack) I. C. Nielsen	Aporusayunnanensis(Pax et Hoffm.) Metc.	Albizia corniculata (Lour.) Druce	Artocarpus styracifolius Pierre
9	Alniphyllum fortunei (Hemsl.) Makino	Castanopsis fargesii Franch.	Archidendron lucidum (Benth.) Nielsen	Callicarpa loboapiculata F. P. Metcalf	Styphnolobium japonicum (L.) Schott	Archidendron clypearia (Jack.) Nielsen	Alniphyllum fortunei (Hemsl.) Makino	Beilschmiedia fordii Dunn
10	Antidesma japonicum Sieb. et Zucc.	Cinnamomum appelianum Schewe	Ardisia hanceana Mez	Camellia crapnelliana Tutcher	Cotinus coggygria var. cinereus Engl.	BauhiniabrachycarpaWall.	Anneslea fragrans Wall.	Betula luminifera H. Winkl.
11	Aphanamixis grandifolia Bl.	Cinnamomum liangii Allen	Ardisia quinquegona Bl.	Camellia fraterna Hance	Buxus sinica (Rehder & E. H. Wilson) M. Cheng	Beilschmiedia delicata S. Lee et Y. T. Wei	Antidesma japonicum Sieb. et Zucc.	Boehmeria nivea (Linn.) Gaudich.
12	Aporusa yunnanensis (Pax et Hoffm.) Metc.	Clethra delavayi Franch.	Artocarpus styracifolius Pierre	Camellia mairei (H. Lév.) Melch.	Fraxinus insularis Hemsl.	Boehmeriatricuspis(Hance) Makino	Aralia armata (Wall.) Seem.	Callicarpa macrophylla Vahl
13	Archidendron clypearia (Jack.) Nielsen	Cleyera pachyphylla Chun ex H. T. Chang	Beilschmiedia delicata S. Lee et Y. T. Wei	Camellia pitardii Cohen-Stuart	Coriaria nepalensis Wall.	Boniodendron minus (Hemsl.) T. Chen	Betula luminifera H. Winkl.	Castanopsis fabri Hance
14	Archidendron lucidum (Benth.) Nielsen	Cyclobalanopsis championi (Benth.) Oerst.	Blastus cochinchinensis Lour.	Camphora foveolata (Merr.) Y. Yang, Bing Liu & Zhi Yang	Lespedeza thunbergii subsp. formosa (Vogel) H. Ohashi	Brassaiopsis glomerulata (Bl.) Regel	Camellia oleifera Abel	Castanopsis fargesii Franch.
15	Ardisia quinquegona Bl.	Daphniphyllum macropodium Miq.	Bridelia pubescens Kurz	Castanopsis eyrei (Champ. ex Benth.) Tutcher	Buddleja officinalis Maxim.	BrassaiopsisgracilisHand.-Mazz.	Camellia sinensis (Linn.) O. Kuntze	Castanopsis fordii Hance
16	Artocarpus styracifolius Pierre	Daphniphyllum oldhamii (Hemsl.) Rosenth.	Camellia sinensis (Linn.) O. Kuntze	Castanopsis faberi Hance	Schima superba Gardner & Champ.	Bridelia balansae Tutcher	Carpinus viminea Wall. ex Lindl.	Castanopsis hystrix A. DC.
17	Aucuba chinensis Benth. var. angusta Wang	Dendropanax dentigerus (Harms) Merr.	Canarium album (Lour.) Raeusch.	Castanopsis fargesii Franch.	Quercus glauca Thunb.	Bridelia fordii Hemsl.	Castanopsis carlesii (Hemsl.) Hayata	Choerospondias axillaris (Roxb.) Burtt. et Hill.
18	Bennettiodendron leprosipes (Clos) Merr.	Diospyros morrisiana Hance	Carallia longipes Chun ex W. C. Ko	Chengiodendron matsumuranum (Hayata) C. B. Shang, X. R. Wang, Yi F. Duan & Yong F. Li	Pteroceltis tatarinowii Maxim.	Broussonetia papyrifera (Linn.) L'Hert. ex Vent.	Castanopsis eyrei (Champ.) Tutch.	Cinnamomum parthenoxylon (Jack) Meisn.
19	Blastus cochinchinensis Lour.	Diplospora dubia (Lindl.) Masam	Caryota maxima Blune	Cinnamomum burmanni (Nees & T. Nees) Blume	Firmiana simplex (L.) W. Wight	Buddleja officinalis Maxim.	Castanopsis fabri Hance	Cinnamomum validinerve Hance
20	Blastus dunnianus Lévl.	Distylium elaeagnoides H. T. Chang	Castanopsis carlesii (Hemsl.) Hayata	Cinnamomum wilsonii Gamble	Morus alba L.	BuddlejadavidiiFranch.	Castanopsis fargesii Franch.	Cinnamomum wilsonii Gamble
21	Bridelia balansae Tutcher	Elaeocarpus duclouxii Gagnep.	Castanopsis fabri Hance	Clethra kaipoensis H. Lév.	Malus baccata (L.) Borkh.	Callicarpa macrophylla Vahl	Castanopsis tibetana Hance	Cipadessa cinerascens (Pellegr.) Hand.-Mazz.
22	Canarium album (Lour.) Raeusch.	Elaeocarpus japonicus Sieb. et Zucc.	Castanopsis fissa (Champ. ex Benth.) Rehd. et Wils.	Cleyera japonica Thunberg	Triadica cochinchinensis Lour.	Camellia sinensis (Linn.) O. Kuntze	Catalpa ovata G. Don	Cleistanthus tonkinensis Jabl.
23	Canarium tramdenum Chan Din Dai & G. P. Yakovlev	Engelhardtia roxburghiana Wall.	Castanopsis hystrix A. DC.	Cleyera pachyphylla Chun ex Hung T. Chang	Photinia serratifolia (Desf.) Kalkman	Canthium dicoccum Merr.	Choerospondias axillaris (Roxb.) Burtt. et Hill.	Clerodendrum cyrtophyllum Turcz.
24	Canthium dicoccum Merr.	Enkianthus chinensis Franch.	Cinnamomum burmannii (C. G. & Th. Nees) Bl.	Cornus controversa Hemsl.	Diospyros kaki Thunb.	Celtis sinensis Pers.	Cinnamomum appelianum Schewe	Clethra delavayi Franch.
25	Canthium horridum Bl.	Eriobotrya fragrans Champ. ex Benth.	Cinnamomum liangii Allen	Cornus hongkongensis Hemsl.	Quercus variabilis Blume	Choerospondias axillaris (Roxb.) Burtt. et Hill.	Cinnamomum liangii Allen	Corylopsis multiflora Hance
26	Carallia longipes Chun ex W. C. Ko	Erythroxylum sinense C. Y. Wu	Cinnamomum parthenoxylon (Jack) Meisn.	Dalbergia hancei Benth.	Viburnum cylindricum Buch.-Ham. ex D. Don	Cinnamomum camphora (L.) J.Presl	Cinnamomum wilsonii Gamble	Cryptocarya concinna Hance
27	Castanopsis hystrix A. DC.	Eurya acuminatissima Merr. et Chun	Cinnamomumbejolghota(Buch.-Ham.) Sweet	Daphniphyllum macropodum Miq.	Wendlandia uvariifolia Hance	Cinnamomum parthenoxylon (Jack) Meisn.	Clethra delavayi Franch.	Daphniphyllum oldhamii (Hemsl.) Rosenth.
28	Cinnamomum parthenoxylon (Jack) Meisn.	Eurya alata Kobuski	Clethra bodinieri Lévl.	Dendropanax dentiger (Harms) Merr.	Callicarpa bodinieri H. Lév.	Cipadessa cinerascens (Pellegr.) Hand.-Mazz.	Cordia dichotoma Forst. f.	Diospyros morrisiana Hance
29	Clethra bodinieri Lévl.	Eurya loquaiana Dunn	Cryptocarya chinensis (Hance) Hemsl.	Dendropanax hainanensis (Merr. & Chun) Chun	Keteleeria davidiana (C. E. Bertrand) Beissn.	Cladrastis platycarpa (Maxim.) Makino	Corylopsis multiflora Hance	Elaeocarpus chinensis (Gardn. et Champ.) Hook. f. ex Benth.
30	Cryptocarya chingii Cheng	Ficus chartacea Wall. ex King	Cryptocarya chingii Cheng	Dipentodon sinicus Dunn	Triadica sebifera (L.) Small	Clausena dunniana Lévl.	Cunninghamia lanceolata (Lamb.) Hook.	Elaeocarpus duclouxiiGagnep.
31	Cryptocarya concinna Hance	Ficus variolosa Lindl. ex Benth.	Cryptocarya concinna Hance	Dipentodon sinicus Dunn	Betula alnoides Buch.-Ham. ex D. Don	Clausena excavata Burm. f.	Daphniphyllum oldhamii (Hemsl.) Rosenth.	Elaeocarpus japonicus Sieb. et Zucc.
32	Cylindrokelupha robinsonii (Gagne p.) Kosterm.	Garcinia multiflora Champ. ex Benth.	Cyclobalanopsis saravanensis (A. Camus) Hjelmq.	Eriobotrya cavaleriei (H. Lév.) Rehder	Toona sinensis (Juss.) Roem.	Clausena lansium (Lour.) Skeels	Dendrobenthamia hongkongensis (Hemsl.) Hutch.	Elaeocarpus sylvestris (Lour.) Poir.
33	Daphniphyllum oldhamii (Hemsl.) Rosenth.	Gardenia hainanensis Merr.	DacrycarpusimbricatusdeLaub.	Erythroxylum sinense C. Y. Wu	Cinnamomum camphora (Linn) Presl	CleidionbracteosumGagnep.	Dendropanax hainanensis (Merr. et Chun) Chun	Engelhardtia roxburghiana Wall.
34	Diospyros morrisiana Hance	Gordonia axillaris (Roxb.) Dietrich	Daphniphyllum calycinum Benth.	Erythroxylum sinense C. Y. Wu	Lyonia ovalifolia var. elliptica (Siebold & Zucc.) Hand.-Mazz.	Clerodendrum japonicum (Thunb.) Sweet	Diospyros kaki Thunb. var. silvestris Makino	Erythroxylum sinense C. Y. Wu
35	Eberhardtia aurata (Pierre ex Dubard) Lec.	Hartia villosa (Merr.) Merr.	Daphniphyllum oldhamii (Hemsl.) Rosenth.	Euonymus laxiflorus Blume ex Miq.	Rhus chinensis Mill.	Clerodendrum mandarinorum Diels	Diospyros morrisiana Hance	Euonymus pittosporoidesC. Y. Cheng ex J. S. Ma
36	Ehretia longiflora Champ. ex Benth.	Helicia reticulata W. T. Wang	Diospyros morrisiana Hance	Euonymus myrianthus hemsl.	Morella rubra Lour.	Cocculus laurifolius DC.	Diplospora dubia (Lindl.) Masam	Eurya loquaiana Dunn
37	Elaeocarpus chinensis (Gardn. et Champ.) Hook. f. ex Benth.	Huodendron biaristatum (W. W. Smith) Rehd.	Ehretia longiflora Champ. ex Benth.	Eurya acuminatissima Merr. & Chun	Diospyros kaki var. silvestris Makino	Coelospermum kanehirae Merr.	Distylium elaeagnoides H. T. Chang	Eurya muricata Dunn
38	Elaeocarpus japonicus Sieb. et Zucc.	Ilex elmerrilliana S. Y. Hu	Elaeocarpus atro-punctatus H. T. Chang	Eurya impressinervis Kobuski	Camellia oleifera Abel	Crateva formosensis (Jacobs) B. S. Sun	Distylium myricoides Hemsl.	Exbucklandia tonkinensis (Lec.) Steenis
39	Elaeocarpus nitentifolius Merr. et Chun	Ilex maclurei Merr.	Elaeocarpus chinensis (Gardn. et Champ.) Hook. f. ex Benth.	Eurya saxicola Hung T. Chang	Vernicia fordii (Hemsl.) Airy Shaw	Croton lachnocarpus Benth.	Elaeocarpus duclouxii Gagnep.	Ficus hirta Vahl
40	Elaeocarpus sylvestris (Lour.) Poir.	Ilex subficoidea S. Y. Hu	Elaeocarpus decipiens Hemsl.	Ficus erecta Thunb.	Ulmus pumila L.	CryptocaryacalcicolaH. W. Li	Elaeocarpus japonicus Sieb. et Zucc.	Ficus langkokensis Drake
41	Engelhardtia roxburghiana Wall.	Ilex subficoidea S. Y. Hu	Elaeocarpus duclouxii Gagnep.	Ficus heteromorpha Hemsl.	Liquidambar formosana Hance	Cyclobalanopsis glauca (Thunb.) Oerst.	Engelhardtia roxburghiana Wall.	Ficus variolosa Lindl. ex Benth.
42	Euonymus centidens Lévl.	Ilex triflora Bl.	Elaeocarpus japonicus Sieb. et Zucc.	Heptapleurum heptaphyllum (L.) Y. F. Deng	Platycarya strobilacea Sieb. et Zucc.	Cyclobalanopsis myrsinaefolia (Blume) Oerst.	Euonymus dielsianus Loes. ex Diels	Garcinia multiflora Champ. ex Benth.
43	Eurya ciliata Merr.	Ilex wilsonii Loes.	Elaeocarpus nitentifolius Merr. et Chun	Heptapleurum minutistellatum (Merr. ex H. L. Li) Y. F. Deng	Bothrocaryum controversum	Daphniphyllum calycinum Benth.	Euonymus myrianthus Hemsl.	Glochidion wrightii Benth.
44	Eurya loquaiana Dunn	Illicium angustisepalum A. C. Smith	Elaeocarpus sylvestris (Lour.) Poir.	Ilex angulata Merr. & Chun	Lyonia villosa (Wall. ex C. B. Clarke) Hand.-Mazz.	Daphniphyllum oldhamii (Hemsl.) Rosenth.	Eurya alata Kobuski	Helicia cochinchinensis Lour.
45	Eurya subintegra Kobuski	Itea chinensis Hook. et Arn.	Elaeocarpus varunua Buch.-Ham.	Ilex ficoidea Hemsl.	Mahonia fortunei (Lindl.) Fedde	Decaspermum gracilentum (Hance)	Eurya distichophylla Hemsl.	Helicia reticulata W. T. Wang

						Merr. et Perry		
46	Exbucklandia populnea (R. Br.) R. W. Brown	Laurocerasus phaeosticta (Hance) S. K. Schneid.	Engelhardtia fenzelii Merr.	Ilex formosana Maxim.	YC	Dendrobenthamia angustata (Chun) Fang	Eurya loquaiana Dunn	Huodendron biaristatum (W. W. Smith) Rehd.
47	Ficus langkokensis Drake	Ligustrum lianum Hsu	Engelhardtia roxburghiana Wall.	Ilex suaveolens (H. Lév.) Loes.		Dillenia turbinata Fin. et Gagnep.	Eurya muricata Dunn	Ilex formosana Maxim.
48	Ficus variolosa Lindl. ex Benth.	Lindera kwangtungensis (Liou) Allen	Erythroxylum sinense C. Y. Wu	Ilex szechwanensis Loes.		Diospyros kaki Thunb. var. silvestris Makino	Fagus longipetiolata Seem	Ilex pubescens Hook. et Arn. var. glabra H. T. Chang
49	Ficus tuphapensis Drake	Lithocarpus calophyllus Chun	Euonymus nitidus Benth.	Ilex wilsonii Loes.		DiospyrosdumetorumW. W. Smith	Ficus heteromorpha Hemsl.	Ilex triflora Bl.
50	Garcinia multiflora Champ. ex Benth.	Lithocarpus elizabethae (Tutch.) Rehd.	Eurya acutisepala Hu et L. K. Ling	Lasianthus chinensis (Champ. ex Benth.) Benth.		Elaeocarpus japonicus Sieb. et Zucc.	Fraxinus chinensis Roxb.	Itea chinensis Hook. et Arn.
51	Garcinia oblongifolia Champ. ex Benth.	Lithocarpus floccosus Huang et Y. T. Chang	Eurya alata Kobuski	Lasianthus japonicus Miq.		Erythropsiskwangsiensis(Hsue) Hsue	Gardenia jasminoides Ellis	Lasianthus lancifolius Hook. f.
52	Gardenia jasminoides Ellis	Lithocarpus naiadarum (Hance) Chun	Eurya macartneyi Champ.	Lasianthus japonicus subsp. longicaudus (Hook. f.) C. Y. Wu & H. Zhu		Eurycorymbus cavaleriei (Lévl.) Rehd. et Hand.-Mazz.	Helicia reticulata W. T. Wang	Laurocerasus phaeosticta (Hance) S. K. Schneid.
53	Glochidion puberum (Linn.) Hutch.	Lithocarpus paihengii Chun et Tsiang	Eurya nitida Korthals	Liquidambar formosana Hance		Ficus erecta Thunb.var. beecheyana (Hook. et Arn.) King	Huodendron biaristatum (W. W. Smith) Rehd.	Lindera kwangtungensis (Liou) Allen
54	Glochidion wrightii Benth.	Litsea cubeba (Lour.) Pers.	Eurya patentipila Chun	Lithocarpus corneus (Lour.) Rehder		Ficus fistulosa Reinw ex Bl.	Ilex kwangtungensis Merr.	Lindera metcalfiana Allen
55	Hartia villosa (Merr.) Merr.	Litsea elongata (Wall. ex Nees) Benth. et Hook.f.	Evodia lepta (Spreng.) Merr.	Lithocarpus hancei (Benth.) Rehder		Ficus hispida Linn.f.	Ilex lohfauensis Merr.	Liquidambar formosana Hance
56	Helicia cochinchinensis Lour.	Lyonia ovalifolia (Wall.) Drude	Ficus esquiroliana Lévl.	Lithocarpus megalophyllus Rehder & E. H. Wilson in Sarg.		Ficus nervosa Heyne ex Roth	Ilex memecylifolia Champ. ex Benth.	Lithocarpus harlandii (Hance) Rehd.
57	Helicia longipetiolata Merr. et Chun	Machilus breviflora (Benth.) Hemsl.	Ficus langkokensis Drake	Litsea elongata (Wall. ex Ness) Benth. & Hook. f.		Ficus tinctoria G. Forst. f. subsp. gibbosa (Bl.) Corner	Ilex micrococca Maxim.	Litsea acutivena Hay.
58	Heliciopsis lobata (Merr.) Sleum.	Machilus decursinervis Chun	Ficus variolosa Lindl. ex Benth.	Litsea mollis Hemsl.		FicuscyrtophyllaWall. ex Miq.	Ilex pubescens Hook. et Arn.	Litsea elongata (Wall. ex Nees) Benth. et Hook.f.
59	Heteropanax brevipedicellatus Li	Machilus pauhoi Kanehirn	Fissistigma oldhamii (Hemsl.) Merr.	Litsea pseudoelongata H. Liu		FicusglaberrimaBl.	Ilex rotunda Thunb.	Litsea greenmaniana Allen
60	Huodendron biaristatum (W. W. Smith) Rehd.	Mallotus japonicus (Thunb.) Muell. Arg.	Fissistigma polyanthum (Hook. f. et Thoms) Merr.	Litsea pungens Hemsl.		FicusoligodonMiq.	Ilex triflora Bl.	Litsea lancilimba Merr.
61	Ilex aculeolata Nakai	Manglietia fordiana Oliv.	Fissistigmachloroneurum	Litsea verticillata Hance		GarciniapaucinervisChun et How	IlexchingianaHu et Tang	Macarangahenryi(Pax et Hoffm.) Rehd.
62	Ilex chuniana S. Y. Hu	Metadina trichotoma (Zoll. ex Mor.) Bakh. f.	Fissistigmalatifolium	Machilus breviflora (Benth.) Hemsl.		Gardenia jasminoides Ellis	Itea chinensis Hook. et Arn.	Machilus chinensis (Champ. ex Benth.) Hemsl.
63	Ilex formosana Maxim.	Michelia maudiae Dunn	Flacourtia rukam Zoll. et Mor.	Machilus leptophylla		Gleditsia sinensis Lam.	Laurocerasus phaeosticta (Hance) S. K. Schneid.	Machilus litseifolia S. Lee
64	Ilex micrococca Maxim.	Microtropis fokienensis Dunn	Garcinia multiflora Champ. ex Benth.	Machilus leptophylla Hand.-Mazz.		Glochidion puberum (Linn.) Hutch.	Lindera glauca (Sieb. et Zucc.) Bl.	Machilus thunbergii Sieb. et Zucc.
65	Ilex pubescens Hook. et Arn. var. glabra H. T. Chang	Myrica rubra (Lour.) Sieb. et Zucc.	Glochidion eriocarpum Champ. ex Benth.	Maclura cochinchinensis (Lour.) Corner		Glycosmispentaphylla(Retz.) Correa	Lindera pulcherrima (Wall.) Benth. var. atteuata Allen	Mallotus barbatus (Wall.) Muell. Arg.
66	Ilex tsangii S. Y. Hu	Nageia fleuryi (Hickel) de Laub.	Helicia cochinchinensis Lour.	Mallotus lianus Croizat		Ilex subficoidea S. Y. Hu	Liquidambar formosana Hance	Meliosma fordii Hemsl.
67	IlexbuergeriMiq.	Neolitsea cambodiana Lec.	Helicia reticulata W. T. Wang	Manglietia conifera Dandy		ItoaorientalisHemsl.	Lithocarpus elizabethae (Tutch.) Rehd.	Neolitsea chuii Merr.
68	Itea chinensis Hook. et Arn.	Neolitsea chuii Merr.	Ilex ficoidea Hemsl.	Manglietia fordiana Oliv.		Jatropha curcas Linn.	Lithocarpus naiadarum (Hance) Chun	Neolitseaaurata(Hay.) Koidz. var.chekiangensis(Nakai) Yang et P. H. Huang
69	Itoa orientalis Hemsl.	Neolitsea phanerophlebia Merr.	Ilex kwangtungensis Merr.	Michelia maudiae Dunn		LaurocerasusaustralisYü et Lu	Litsea cubeba (Lour.) Pers.	Pavetta hongkongensis Bremek.
70	Lasianthus appressihirtus Simizu	Osmanthus marginatus (Champ. ex Benth.) Hemsl.	Ilex macrocarpa Oliv.	Michelia mediocris Dandy		Ligustrum sinense Lour.	Litsea elongata (Wall. ex Nees) Benth. et Hook.f.	Photinia beauverdiana Schneid.
71	Lasianthus henryi Hutch.	Parakmeria lotungensis (Chun & C. Tsoong) Y. W. Law	Ilex pubescens Hook. et Arn.	Microtropis tetragona Merr. & F. L. Freeman		Lindera pulcherrima (Wall.) Benth. var. atteuata Allen	Lyonia ovalifolia (Wall.) Drude	Photinia prunifolia (Hook. et Arn.) Lindl.
72	Lasianthus lancifolius Hook. f.	Pentaphylax euryoides Gardn. et Champ.	Ilex szechwanensis Loes.	Miliusa sinensis Finet & Gagnep.		Lirianthe mulunica(Y. W. Law et Q. W. Zeng) N. H. Xia et C. Y. Wu	Macaranga denticulata (Bl.)Muell. Arg.	Polyalthia plagioneura Diels
73	Lindera chunii Merr.	Photinia davidsoniae Rehd. et Wils.	Ilex triflora Bl.	Neolitsea aurata (Hayata) Koidz.		Litsea atrata S. Lee	Machilus chinensis (Champ. ex Benth.) Hemsl.	Pygeum topengii Merr.
74	Lindera metcalfiana Allen	Photinia glabra (Thunb.) Maxim.	Ilex viridis Champ. ex Benth.	Neolitsea shingningensis Yen C. Yang & P. H. Huang		Litsea variabilis Hemsl.	Machilus decursinervis Chun	RapaneakwangsiensisWalker
75	Lindera caudata (Nees) Hook. f.	Pinus massoniana Lamb.	Illicium micranthum Dunn	Neolitsea zeylanica (Nees & T. Nees) Merr.		LuculiapinceanaHook.	Machilus leptophylla Hand.-Mazz.	Rhododendron simsii Planch.
76	Liquidambar formosana Hance	Rapanea neriifolia (Sieb. et Zucc.) Mez	Illicium verum Hook. f.	Photinia glabra (Thunb.) Maxim.		Machilus chekiangensis S. K. Lee	Mallotus apelta (Lour.) Muell. Arg.	Rhodoleia championi Hook.
77	Lithocarpus elizabethae (Tutch.) Rehd.	Raphiolepis ferruginea Metcalf	Itea chinensis Hook. et Arn.	Photinia serratifolia (Desf.) Kalkman		Machilus decursinervis Chun	Manglietia hainanensis Dandy	Schefflera delavayi (Franch.) Harms ex Diels
78	Lithocarpus glabra (Thunb.) Nakai	Raphiolepis indica (Linn.) Lindl.	Lindera kwangtungensis (Liou) Allen	Pieris formosa (Wall.) D. Don		Machilus litseifolia S. Lee	Meliosma glandulosa Cufod.	Schefflera heptaphylla (Linn.) Frodin
79	Lithocarpus litseifolius (Hance) Chun	Rhododendron henryi Hance	Lindera metcalfiana Allen	Pittosporum brevicalyx (Oliv.) Gagnep.		Maesa perlarius (Lour.) Merr.	Meliosma squamulata Hance	Sloanea sinensis (Hance) Hemsl.
80	Litsea elongata (Wall. ex Nees) Benth. et Hook.f.	Rhododendron simiarum Hance	Lithocarpus elizabethae (Tutch.) Rehd.	Pittosporum glabratum Lindl.		Mallotus barbatus (Wall.) Muell. Arg.	Metadina trichotoma (Zoll. ex Mor.) Bakh. f.	Styrax suberifolius Hook. et Arn.
81	Litsea mollis Hemsl.	Rhodoleia championi Hook.	Lithocarpus glabra (Thunb.) Nakai	Pittosporum glabratum var. neriifolium Rehder & E. H. Wilson		Mallotus japonicus (Thunb.) Muell. Arg.	Michelia foveolata Merr. ex Dandy	Symplocos cochinchinensis (Lour.) S. Moore
82	Litsea subcoriacea Yang et P. H. Huang	Schefflera delavayi (Franch.) Harms ex Diels	Litsea acutivena Hay.	Pittosporum podocarpum Gagnep.		Mallotus philippensis (Lam.) Muell. Arg.	Michelia maudiae Dunn	Symplocos congesta Benth.
83	Litsea variabilis Hemsl.	Schima argentea Pritz ex Diels	Litsea greenmaniana Allen	Pittosporum trigonocarpum H. Lév.		Micromelum integerrimum (Buch.-Ham.) Wight & Arn.	Michelia skinneriana Dunn	Symplocos lancifolia Sieb. et Zucc.
84	Machilus chinensis (Champ. ex Benth.) Hemsl.	Schoepfia chinensis Gardn. et Champ.	Macaranga denticulata (Bl.)Muell. Arg.	Prunus spinulosa Siebold & Zucc.		Miliusa sinensis Finet et Gagnep.	Microtropis fokienensis Dunn	Symplocos wikstroemiifolia Hayata
85	Machilus litseifolia S. Lee	Skimmia arborescens T. Anders. ex. Gamble	Machilus chinensis (Champ. ex Benth.) Hemsl.	Prunus undulata Buch.-Ham. ex D. Don		Murrayapaniculata(L.) Jack.	Mussaenda esquirolii Lévl.	Syzygium buxifolium Hook. et Arn.
86	Machilus salicina Hance	Skimmia arborescens T. Anders. ex. Gamble	Machilus ichangensis Rehd. et Wils.	Pterostyrax psilophyllus Diels ex Perkins		Nothapodytes pittosporoides (Oliv.) Sleum.	Myrica rubra (Lour.) Sieb. et Zucc.	Syzygium rehderianum Merr. et Perry
87	Machilus thunbergii Sieb. et Zucc.	Sorbus folgneri (Schneid.) Rehd.	Machilus leptophylla Hand.-Mazz.	Quercus jenseniana Hand.-Mazz.		Oreocnide frutescens (Thunb.) Miq.	Neolitsea aurata (Hay.) Koidz. var. paraciculata (Nakai) Yang et P. H. Huang	Toxicodendron succedaneum (Linn.) O. Kuntze
88	Machilus velutina Champ. ex Benth.	Styrax odoratissimus Champ. ex Benth.	Machilus litseifolia S. Lee	Reevesia pubescens Mast.		OreocnidekwangsiensisHand.-Mazz.	Neolitsea levinei Merr.	Trichiliaconnaroides(Wight et Arn.) Bentv.
89	Madhuca pasquieri (Dubard) Lam.	Symplocos adenopus Hance	Machilus pauhoi Kanehirn	Rehderodendron kwangtungense Chun		Pavetta hongkongensis Bremek.	Neolitsea phanerophlebia Merr.	Tsoongiodendron odorum Chun
90	Maesa perlarius (Lour.) Merr.	Symplocos confusa Brand.	Macrosolen cochinchinensis (Lour.) Van Tiegh.	Rehderodendron kwangtungense Chun		Photinia parvifolia (Pritz.) Schneid.	Nyssa sinensis Oliv.	Turpinia arguta (Lindl.) Seem.
91	Meliosma fordii Hemsl.	Symplocos decora Hance	Maesa perlarius (Lour.) Merr.	Rehderodendron macrocarpum Hu		Photinia villosa (Thunb.) DC. var. sinica Rehd. et Wils.	Osmanthus attenuatus P. S. Green	Vaccinium bracteatum Thunb.
92	Meliosma thorelii Lecomte	Symplocos lancifolia Sieb. et Zucc.	Medinilla septentrionalis (W. W. Smith) H. L. Li	Rhododendron cavaleriei H. Lév.		Phyllanthus urinaria Linn.	Pentaphylax euryoides Gardn. et Champ.	Vaccinium trichocladum Merr. et Metc.
93	Melodinus fusiformis Champ. ex Benth.	Symplocos laurina (Retz.) Wall.	Meliosma fordii Hemsl.	Rhododendron latoucheae Franch.		Picrasma quassioides (D. Don) Benn.	Photinia beauverdiana Schneid.	Vernicia fordii (Hemsl.) Airy Shaw

94	Metadina trichotoma (Zoll. ex Mor.) Bakh. f.	Symplocos wikstroemiifolia Hayata	Meliosma thorelii Lecomte	Rhododendron simiarum Hance	Pittosporum tobira (Thunb.) Ait.	Photinia glabra (Thunb.) Maxim.	Viburnum odoratissimum Ker-Gawl.
95	Michelia maudiae Dunn	Syzygium buxifolium Hook. et Arn.	Metadina trichotoma (Zoll. ex Mor.) Bakh. f.	Rhodoleia championii Hook. f.	PittosporumtonkinenseGagnep.	Pinus massoniana Lamb.	
96	Myrica rubra (Lour.) Sieb. et Zucc.	Ternstroemia gymnanthera (Wight et Arn.) Beddome	MicheliafloribundaFinet et Gagn.	Rhoiptelea chiliantha Diels & Hand.-Mazz.	PlatycaryalongipesWu	Pittosporum pauciflorum Hook. et Arn.	
97	Neolitsea pulchella (Meissn.) Merr.	Toxicodendron succedaneum (Linn.) O. Kuntze	Myrica rubra (Lour.) Sieb. et Zucc.	Sassafras tzumu (Hemsl.) Hemsl.	Pouzolziasanguinea(Bl.) Merr.	Polygala fallax Hemsl.	
98	Neolitsea aurata (Hay.) Koidz. var. chekiangensis (Nakai) Yang et P. H. Huang	Vaccinium bracteatum Thunb.	Neolitsea chuii Merr.	Schima argentea E. Pritz.	Pterocarpus indicus Willd.	Premna microphylla Turcz.	
99	Ormosia semicastrata Hance f. lichiifolia How		Nyssa sinensis Oliv.	Schima superba Gardner & Champ.	Pterospermum heterophyllum Hance	Rapanea neriifolia (Sieb. et Zucc.) Mez	
100	Parakmeria lotungensis (Chun & C. Tsoong) Y. W. Law		Pavetta hongkongensis Bremek.	Sorbus aronioides Rehder in Sarg.	Radermachera sinica (Hance) Hemsl.	Raphiolepis indica (Linn.) Lindl.	
101	Polyalthia plagioneura Diels		Photinia prunifolia (Hook. et Arn.) Lindl.	Styrax tonkinensis (Pierre) Craib ex Hartwich	Rapanea neriifolia (Sieb. et Zucc.) Mez	Rhododendron bachii Lévl.	
102	Psychotria asiatica Linn.		Polyalthia plagioneura Diels	Symplocos adenopus Hance	RapaneakwangsiensisWalker	Rhododendron cavaleriei Lévl.	
103	Pygeum topengii Merr.		Psychotria asiatica Linn.	Symplocos anomala Brand	RhamnussubapetalaMerr.	Rhododendron kwangtungense Merr. et Chun	
104	Rapanea neriifolia (Sieb. et Zucc.) Mez		Pygeum topengii Merr.	Symplocos lucida (Thunb.) Siebold & Zucc.	Rhapis multifida Burret	Rhododendron moulmainense Hook.	
105	Raphiolepis indica (Linn.) Lindl.		Rapanea neriifolia (Sieb. et Zucc.) Mez	Symplocos prunifolia Siebold & Zucc.	Rubovietnamia aristataTirveng.	Sapium discolor (Champ. ex Benth.) Muell. Arg.	
106	Reevesia longipetiolata Merr. et Chun		Sapium discolor (Champ. ex Benth.) Muell. Arg.	Symplocos ramosissima Wall. ex G. Don	Sauropus reticulatus X. L. Mo ex P. T. Li	Sassafras tzumu (Hemsl.) Hemsl.	
107	Rhododendron henryi Hance		Saurauia tristyla DC.	Symplocos sumuntia Buch.-Ham. ex D. Don	Schefflera heptaphylla (Linn.) Frodin	Schefflera delavayi (Franch.) Harms ex Diels	
108	Rhododendron latoucheae Franch.		Schefflera heptaphylla (Linn.) Frodin	Symplocos theophrastifolia Siebold & Zucc.	Sinoadina racemosa (Sieb. et Zucc.) Ridsd.	Schefflera minutistellata Merr. ex Li	
109	Rhodomyrtus tomentosa (Ait.) Hassk.		Schima superba Gardn. et Champ.	Symplocos wikstroemiifolia Hayata	Sinosideroxylon wightianum (Hook. et Arn.) Aubrn.	Schima wallichii (DC.) Korthals	
110	Sarcosperma laurinum (Benth.) Hook. f.		Styrax confusus Hemsl.	Ternstroemia gymnanthera (Wight & Arn.) Bedd.	Sterculia lanceolata Cav.	Sloanea sinensis (Hance) Hemsl.	
111	Schefflera heptaphylla (Linn.) Frodin		Styrax odoratissimus Champ. ex Benth.	Ternstroemia kwangtungensis Merr.	Sterculia nobilis Smith	Styrax faberi Perk.	
112	Schima superba Gardn. et Champ.		Symplocos adenophylla Wall.	Ternstroemia luteoflora L. K. Ling	SterculiaeuosmaW. W. Smith	Styrax suberifolius Hook. et Arn.	
113	Sterculia lanceolata Cav.		Symplocos glauca (Thunb.) Koidz.	Viburnum sempervirens K. Koch	Syzygium levinei (Merr.) Merr.	Styrax tonkinensis (Pierre) Craib. ex Hartw.	
114	Symplocos adenophylla Wall.		Symplocos pseudobarberina Gontsch.	Zanthoxylum dissitum Hemsl.	SyzygiumchunianumMerr. et Perry	Symplocos congesta Benth.	
115	Symplocos anomala Brand		Symplocos sumuntia Buch.-Ham. ex D. Don		Tarenna depauperata Hutchins.	Symplocos lancifolia Sieb. et Zucc.	
116	Symplocos congesta Benth.		Syzygium rehderianum Merr. et Perry		Tirpitziasinensis(Hemsl.) Hallier	Symplocos pseudobarberina Gontsch.	
117	Symplocos heishanensis Hayata		ToonaciliataRoem.		Toxicodendron succedaneum (Linn.) O. Kuntze	Symplocos stellaris Brand	
118	Symplocos lancifolia Sieb. et Zucc.		Toxicodendron sylvestris (Sieb. et Zucc.) Tardieu		Turpinia montana (Bl.) Kurz.	Symplocos subconnata Hand.-Mazz.	
119	Symplocos laurina (Retz.) Wall.		Trichiliaconnaroides(Wight et Arn.) Bentv.		Ulmus parvifolia Jacq.	Symplocos wikstroemiifolia Hayata	
120	Syzygium hancei Merr. et Perry		Vitex quinata (Lour.) Will.		Vitex quinata (Lour.) Will.	SymplocospoilaneiGuill.	
121	Syzygium odoratum (Lour.) DC.		Wendlandia uvariifolia Hance		Walsura yunnanensis C. Y. Wu	Syzygium buxifolium Hook. et Arn.	
122	Syzygium imitans Merr. et Perry		Xanthophyllum hainanense Hu		Wrightia pubescens R. Br.	Ternstroemia gymnanthera (Wight et Arn.) Beddome	
123	Toxicodendron succedaneum (Linn.) O. Kuntze				Xylosma controversum Clos	Toxicodendron succedaneum (Linn.) O. Kuntze	
124	Trichiliaconnaroides(Wight et Arn.) Bentv.				Xylosma racemosum (Sieb. et Zucc.) Miq.	Turpinia arguta (Lindl.) Seem.	
125	Tsoongia axillariflora Merr.				Zanthoxylum armatum DC.	Vaccinium bracteatum Thunb.	
126	Tsoongiodendron odorum Chun				Zenia insignis Chun	Vernicia montana Lour.	
127	Wendlandia uvariifolia Hance				ZiziphusincurvaRoxb.		
128	Wikstroemia indica (Linn.) C. A. Mey.						
129	Xanthophyllum hainanense Hu						
