# Supplementary material for: Species and structural diversity of trees at the structural type level
Source: BMC Ecol Evol. 2024 Mar 28;24:40. doi: 10.1186/s12862-024-02229-y (PMC10976781; doi:10.1186/s12862-024-02229-y)
Supplement: Supplementary file 2 — Supplementary Material 2. [file 12862_2024_2229_MOESM2_ESM.docx]

**Table S2**: Distribution pattern of structural types

| Site | ReT/_Wi=0.0_ | | | ReT/_Wi=0.25_ | | | RaT/_Wi=0.5_ | | | CT/_Wi=0.75_ | | | CT/_Wi=1.0_ | | |
| --- | --- | --- | --- | --- | --- | --- | --- | --- | --- | --- | --- | --- | --- | --- | --- |
|  | $\bar{w}_{0.0}$ | Range | Distribution pattern | $\bar{w}_{0.25}$ | Range | Distribution pattern | $\bar{w}_{0.5}$ | Range | Distribution pattern | $\bar{w}_{0.75}$ | Range | Distribution pattern | $\bar{w}_{1.0}$ | Range | Distribution pattern |
| CWLS1 | 0.530 | [0414,0.586] | Random | 0.536 | [0.485,0.515] | Cluster | 0.519 | [0.491,0.509] | Cluster | 0.551 | [0.485,0.515] | Cluster | 0.542 | [0.472,0.528] | Cluster |
| CWLS2 | 0.556 | [0.400,0.600] | Random | 0.521 | [0.481,0.519] | Cluster | 0.524 | [0.489,0.511] | Cluster | 0.559 | [0.482,0.518] | Cluster | 0.545 | [0.464,0.536] | Cluster |
| CWLS3 | 0.656 | [0.394,0.606] | Cluster | 0.534 | [0.483,0.517] | Cluster | 0.522 | [0.490,0.510] | Cluster | 0.543 | [0.482,0.518] | Cluster | 0.555 | [0.472,0.528] | Cluster |
| DMS1 | 0.565 | [0.418,0.582] | Random | 0.518 | [0.485,0.515] | Cluster | 0.524 | [0.492,0.508] | Cluster | 0.546 | [0.485,0.515] | Cluster | 0.667 | [0.482,0.518] | Cluster |
| DMS2 | 0.567 | [0.416,0.584] | Random | 0.541 | [0.486,0.514] | Cluster | 0.536 | [0.493,0.507] | Cluster | 0.560 | [0.486,0.514] | Cluster | 0.668 | [0.485,0.515] | Cluster |
| DMS3 | 0.500 | [0.424,0.576] | Random | 0.548 | [0.486,0.514] | Cluster | 0.536 | [0.492,0.508] | Cluster | 0.557 | [0.485,0.515] | Cluster | 0.674 | [0.485,0.515] | Cluster |
| DYS1 | 0.554 | [0.386,0.614] | Random | 0.523 | [0.483,0.517] | Cluster | 0.533 | [0.490,0.510] | Cluster | 0.549 | [0.482,0.518] | Cluster | 0.917 | [0.486,0.514] | Cluster |
| DYS2 | 0.544 | [0.391,0.603] | Random | 0.536 | [0.484,0.516] | Cluster | 0.520 | [0.491,0.509] | Cluster | 0.556 | [0.483,0.517] | Cluster | 0.642 | [0.477,0.523] | Cluster |
| DYS3 | 0.500 | [0.424,0.576] | Random | 0.548 | [0.486,0.514] | Cluster | 0.536 | [0.492,0.508] | Cluster | 0.557 | [0.485,0.515] | Cluster | 0.674 | [0.485,0.515] | Cluster |
| DYS4 | 0.553 | [0.430,0.570] | Random | 0.512 | [0.485,0.515] | Random | 0.511 | [0.491,0.509] | Cluster | 0.545 | [0.484,0.516] | Cluster | 0.575 | [0.472,0.528] | Cluster |
| HP2 | 0.639 | [0.359,0.641] | Random | 0.541 | [0.482,0.518] | Cluster | 0.542 | [0.491,0.509] | Cluster | 0.577 | [0.481,0.519] | Cluster | 0.711 | [0.482,0.518] | Cluster |
| HP3 | 0.650 | [0.390,0.610] | Cluster | 0.539 | [0.484,0.516] | Cluster | 0.561 | [0.492,0.508] | Cluster | 0.575 | [0.483,0.517] | Cluster | 0.829 | [0.489,0.511] | Cluster |
| HP4 | 0.573 | [0.413,0.587] | Random | 0.537 | [0.487.0.513] | Cluster | 0.533 | [0.493,0.507] | Cluster | 0.566 | [0.485,0.515] | Cluster | 0.692 | [0.484,0.516] | Cluster |
| HP5 | 0.586 | [0.427,0.573] | Cluster | 0.531 | [0.486,0.514] | Cluster | 0.541 | [0.493,0.507] | Cluster | 0.568 | [0.485,0.515] | Cluster | 0.721 | [0.486,0.514] | Cluster |
| JWS1 | 0.500 | [0.390,0.610] | Random | 0.547 | [0.480,0.520] | Cluster | 0.540 | [0.489,0.511] | Cluster | 0.576 | [0.479,0.521] | Cluster | 0.691 | [0.479,0.521] | Cluster |
| JWS2 | 0.500 | [0.378,0.622] | Random | 0.535 | [0.484,0.516] | Cluster | 0.529 | [0.491,0.509] | Cluster | 0.533 | [0.482,0.518] | Cluster | 0.688 | [0.481,0.519] | Cluster |
| JWS3 | 0.531 | [0.351,0.649] | Random | 0.543 | [0.485,0.515] | Cluster | 0.549 | [0.492,0.508] | Cluster | 0.567 | [0.484,0.516] | Cluster | 0.759 | [0.488,0.512] | Cluster |
| JWS4 | 0.605 | [0.402,0.598] | Cluster | 0.569 | [0.486,0.514] | Cluster | 0.558 | [0.493,0.507] | Cluster | 0.566 | [0.485,0.515] | Cluster | 0.805 | [0.490,0.510] | Cluster |
| ML1 | 0.577 | [0.382,0.618] | Random | 0.552 | [0.480,0.520] | Cluster | 0.530 | [0.489,0.511] | Cluster | 0.562 | [0.478,0.522] | Cluster | 0.898 | [0.486,0.514] | Cluster |
| ML2 | 0.700 | [0.312,0.688] | Cluster | 0.543 | [0.481,0.519] | Cluster | 0.555 | [0.491,0.509] | Cluster | 0.560 | [0.478,0.522] | Cluster | 0.808 | [0.486,0.514] | Cluster |
| ML3 | 0.625 | [0.413,0.587] | Cluster | 0.526 | [0.484,0.516] | Cluster | 0.529 | [0.491,0.509] | Cluster | 0.556 | [0.482,0.518] | Cluster | 0.903 | [0.488,0.512] | Cluster |
| ML4 | 0.588 | [0.397,0.603] | Random | 0.514 | [0.482,0.518] | Random | 0.542 | [0.490,0.510] | Cluster | 0.544 | [0.480,0.520] | Cluster | 0.866 | [0.485,0.515] | Cluster |
| ML5 | 0.559 | [0.397,0.603] | Random | 0.513 | [0.482,0.518] | Random | 0.524 | [0.490,0.510] | Cluster | 0.529 | [0.480,0.520] | Cluster | 0.794 | [0.478,0.522] | Cluster |
| SWS2 | 0.587 | [0.411,0.589] | Random | 0.526 | [0.487.0.513] | Cluster | 0.518 | [0.493,0.507] | Cluster | 0.536 | [0.487,0.513] | Cluster | 0.772 | [0.482,0.518] | Cluster |
| SWS4 | 0.554 | [0.437,0.563] | Random | 0.508 | [0.489,0.511] | Random | 0.519 | [0.493,0.507] | Cluster | 0.534 | [0.488,0.512] | Cluster | 0.791 | [0.485,0.515] | Cluster |
| SWS5 | 0.564 | [0.437,0.563] | Cluster | 0.519 | [0.489,0.511] | Cluster | 0.515 | [0.493,0.507] | Cluster | 0.536 | [0.488,0.512] | Cluster | 0.654 | [0.482,0.518] | Cluster |
| YC1 | 0.589 | [0.386,0.614] | Random | 0.519 | [0.481,0.519] | Cluster | 0.513 | [0.489,0.511] | Cluster | 0.501 | [0.481,0.519] | Random | 0.568 | [0.471,0.529] | Cluster |
| YC2 | 0.604 | [0.378,0.622] | Random | 0.529 | [0.477,0.523] | Cluster | 0.517 | [0.486,0.514] | Cluster | 0.547 | [0.476,0.524] | Cluster | 0.538 | [0.460,0.540] | Random |
| YC3 | 0.813 | [0.351,0.649] | Cluster | 0.512 | [0.481,0.519] | Random | 0.550 | [0.489,0.511] | Cluster | 0.566 | [0.479,0.521] | Cluster | 0.587 | [0.478,0.522] | Cluster |
| YC4 | 0.568 | [0.409,0.591] | Random | 0.499 | [0.477,0.523] | Random | 0.509 | [0.486,0.514] | Random | 0.532 | [0.475,0.525] | Cluster | 0.538 | [0.452,0.548] | Random |
| YC5 | 0.583 | [0.328,0.672] | Random | 0.525 | [0.470,0.530] | Random | 0.498 | [0.482,0.518] | Random | 0.576 | [0.466,0.534] | Cluster | 0.570 | [0.455,0.555] | Cluster |
| YC6 | 0.529 | [0.416,0.584] | Random | 0.523 | [0.485,0.515] | Cluster | 0.519 | [0.491,0.509] | Cluster | 0.535 | [0.484,0.516] | Cluster | 0.589 | [0.474,0.526] | Cluster |
| YC7 | 0.576 | [0.442,0.558] | Cluster | 0.508 | [0.488,0.512] | Random | 0.508 | [0.493,0.507] | Cluster | 0.523 | [0.487,0.513] | Cluster | 0.552 | [0.475,0.525] | Cluster |
| YC8 | 0.578 | [0.440,0.560] | Cluster | 0.509 | [0.489,0.511] | Random | 0.522 | [0.494,0.506] | Cluster | 0.550 | [0.487,0.513] | Cluster | 0.751 | [0.488,0.512] | Cluster |
